# Integrating interconception care in preventive child health care services: The Healthy Pregnancy 4 All program

**DOI:** 10.1371/journal.pone.0224427

**Published:** 2019-11-06

**Authors:** Meertien K. Sijpkens, Jacqueline Lagendijk, Minke R. C. van Minde, Marlou L. A. de Kroon, Loes C. M. Bertens, Ageeth N. Rosman, Eric A. P. Steegers

**Affiliations:** 1 Department of Obstetrics and Gynaecology, Division of Obstetrics and Prenatal Medicine, Erasmus MC, University Medical Center Rotterdam, Rotterdam, The Netherlands; 2 Department of Public Health, Erasmus MC, University Medical Center Rotterdam, Rotterdam, the Netherlands; 3 Department of Health Sciences, University Medical Center Groningen, Groningen, The Netherlands; 4 Department of Health Care Studies, Rotterdam University of Applied Sciences, Rotterdam, The Netherlands; University of Ghana College of Health Sciences, GHANA

## Abstract

**Background:**

Most parents with young children pay routine visits to Well-Baby Clinics, or so-called Preventive Child Health Care (PCHC) services. This offers a unique opportunity to promote and deliver interconception care. This study aimed to integrate such care and perform an implementation evaluation.

**Methods:**

In seven Dutch municipalities, PCHC professionals were instructed to discuss the possibility of an interconception care consultation during each routine six-months well-baby visit. The primary outcome of this study was coverage of the intervention, quantified as the proportion of visits during which women were informed about interconception care. Secondary outcomes included adoption, fidelity, feasibility, appropriateness, acceptability and effectiveness of the intervention, studied by surveying PCHC professionals and women considering becoming pregnant.

**Results:**

The possibility of interconception care was discussed during 29% (n = 1,849) of all visits, and 60% of the PCHC physicians adopted the promotion of interconception care by regularly informing women. About half of the PCHC professionals and most women judged integration of interconception care in PCHC appropriate and acceptable. Estimated feasibility was poor, since 13% of the professionals judged future integration in daily practice as probable. The uptake of interconception care consultations was low (n = 4 consultations).

**Conclusions:**

Promotion of interconception care was achieved in approximately one-third of the routine PCHC consultations and appeared promising with regards to adoption, appropriateness and acceptability. However, concerns on feasibility and uptake of interconception care consultations in daily practice remain. Suggestions for improvement may include further integration of interconception care health promotion in routine PCHC consultations, while allocating sufficient resources.

## Introduction

Well-Baby Clinics, otherwise known as Preventive Child Health Care (PCHC, Box 1) services, provide unique access to women between pregnancies. Most women with young children go to routinely scheduled PCHC appointments, which offers an opportunity for interconception care (ICC). ICC is a type of preconception care (PCC) between pregnancies, aiming to optimize parental health prior to pregnancy [1]. Currently, PCC and ICC services reach only a few women and antenatal care often starts too late to prevent the occurrence of risk factors during the periconception period which affect pregnancy outcomes [2, 3]. Many periconception risk factors are associated with the course of pregnancy and with child health outcomes [4–6], including behavioral, medical, and psychosocial risks [7]. These risk factors are frequent among women who may become pregnant, and certain groups of women in particular need extra attention in preventive preconception strategies [8, 9]. For instance, large socio-economic inequalities exist in prevalence of risk factors such as smoking and inadequate folic acid intake [8, 10–12]. In addition, some studies suggest that these specific risk factors are also more prevalent in parous women [13, 14]. Besides, parous women may exhibit risks for recurrence of adverse pregnancy outcomes, such as preterm birth and fetal growth restriction. ICC could address such risks, but delivery and uptake of both PCC and ICC remain scarce [15, 16].

The idea that PCHC providers could contribute to the provision of ICC has been previously recognized in an advisory report on preconception care drafted by the Health Council of the Netherlands [17]. Until recently, a few promising ICC intervention studies focusing on folic acid supplementation were conducted in both Dutch and international PCHC settings [18, 19]. But to our knowledge, strategies to integrate more comprehensive ICC in PCHC are uncommon. We hypothesized that PCHC providers could promote and deliver comprehensive ICC consultations to increase the uptake of ICC and to improve preconceptional health. To understand how ICC could work in the real time practice of PCHC, implementation research is essential [20]. This study aimed to implement and evaluate promotion and delivery of ICC in PCHC centers in the Netherlands.

## Methods

### Setting

The study was embedded in the HP4All-2 program [21]. The HP4All programs aim to improve maternal and perinatal health by enhancing risk-guided care from the preconception period through to the interconception period [21, 22]. In the preceding HP4All-1 program, recruitment for and delivery of PCC at general practitioners (GPs) and midwifery practices was employed, which included some PCHC services distributing information leaflets about PCC [23, 24]. In total, 587 applications for PCC consultations were registered, mostly (n = 424) prompted by the 132,129 invitation letters from municipalities and general practitioners [24]. Only 6 consultations resulted from leaflets distributed at PCHC services. The HP4All-2 program proposed to enhance the role of PCHC services and focused specifically on ICC. Both programs have intended to reduce perinatal health inequalities by focusing on municipalities with higher rates of adverse pregnancy outcomes than the national average [21, 22]. The current study was conducted in seven municipalities where, together with local government representatives, cooperation was sought with the PCHC services (Box 1)[21].

**Box 1. Preventive Child Health Care in the Netherlands**

The organization of Preventive Child Health Care (PCHC) in the Netherlands has some distinct characteristics [25–27]. It is organized nationally, but formalized on the municipal level. PCHC teams, consisting of trained physicians and nurses, monitor and promote optimal growth and development of the child by providing immunizations, screenings and health advice. If needed, they refer directly to general practitioners or pediatricians. PCHC is offered free of charge to all children from birth until the age of nineteen years. The care for children up to the age of four years is organized along a standard set of consultations in local well-baby clinics, which have high (>95%) attendance rates [18].

### Intervention

**Fig 1:**
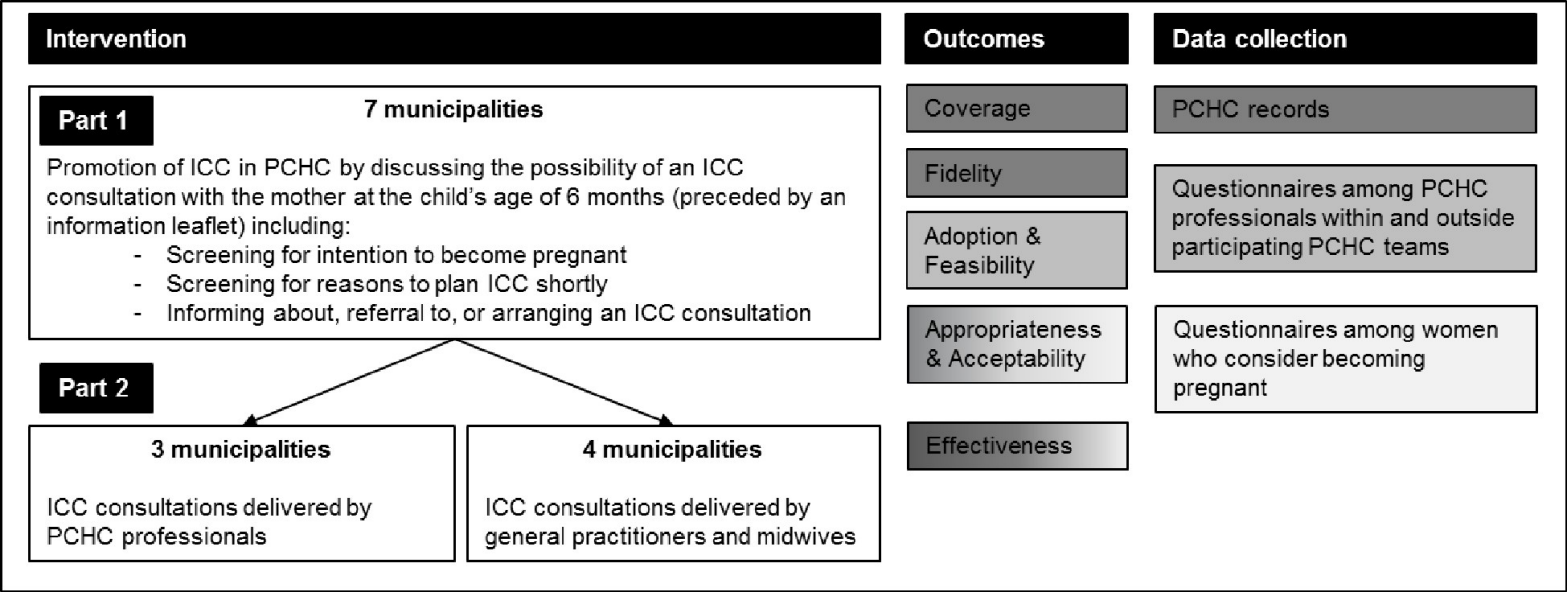
Outline of the study.

The ICC intervention consisted of two-parts (Fig 1), of which the first part was applied the same way across the seven municipalities, while the second part could differ per municipality. In the first part of the intervention, promotion of ICC was integrated in routine well-baby consultations at the child’s age of six months, referred to as the ‘six-months consultation’. Promotion consisted of the PCHC physicians screening the mother for her intention to become pregnant in the future, while discussing the possibility of a separate ICC consultation free of charge (see second part of the intervention). In addition, when women considered becoming pregnant, they were screened for the following reasons to direct these women to an ICC consultation at short notice: 1) currently trying to become pregnant, and 2) an obstetrical history of an adverse perinatal outcome (e.g. preterm birth, low birth weight, congenital abnormalities, neonatal asphyxia). Following the promotion of ICC, women could themselves make an appointment for an ICC consultation. For the delivery of these ICC consultations, constituting the second part of the intervention, two different approaches were implemented (Fig 1): in three out of seven participating municipalities PCHC professionals provided ICC consultations themselves; in the other four municipalities PCHC teams referred to a GP or community midwife for an ICC consultation.

### Implementation strategy

In preparation of the implementation of the intervention, a previously performed and published analysis of its possible determinants was taken into consideration. This study was based on four focus group discussions with in total 33 stakeholders representing different backgrounds (i.e. PCHC physicians, PCHC nurses, pediatricians, gynecologists, midwives, GPs, and policymakers)[28]. Barriers that emerged from this study included the lack of consensus about how ICC should be organized, possible financial and logistical barriers in PCHC services that are normally organized around child care instead of maternal care, and municipal differences in PCHC services. Another important expected barrier was the anticipated unfamiliarity with ICC among PCHC professionals and the target group of women who consider becoming pregnant again [28]. Based on this study we provided several educational sessions and accompanying materials prior to the start of this intervention study. The educational sessions, offered to PCHC teams in all municipalities, consisted of a theoretical background lecture on the importance of ICC, and an interactive session with skills training in discussing ICC and pregnancy intention. In a separate session, the logistics of the study were explained. Accompanying materials included information on ICC for the healthcare professionals, as well as information leaflets for women about ICC and what they could expect at the routine six-months consultation. In addition to the provided materials by our research team, one municipality developed a short promotional video, of which the link was sent to women who indicated to consider becoming pregnant. Lastly, during the project, one or two evaluation sessions were planned per PCHC team.

### Participants

The main targets of the intervention were PCHC professionals and women who may become pregnant again.

The integration of ICC among PCHC professionals was studied in two subgroups. The first subgroup consisted of all PCHC physicians and nurses from the teams that were involved in the intervention; and the second consisted of a corresponding number of PCHC physicians and nurses from teams that were not involved in the HP4All programs, serving as a reference group.

All women who visited PCHC teams involved in the HP4All-2 program for the six-months consultation were eligible for the intervention. Additionally, in the first four municipalities that started the intervention (i.e. two of each ICC delivery approach; Fig 1), women who considered becoming pregnant were invited to participate in a questionnaire study if they met the inclusion criteria (i.e. age >18 years and sufficient understanding of the Dutch or English language).

### Outcomes

An overview of all outcome measures is presented in S1 Table. The primary outcome of the study was *coverage* of the intervention, defined as the percentage of regular PCHC six-months consultations in which the possibility of an ICC consultation was discussed [20]. Secondary outcomes included the following other implementation outcomes: *Fidelity*, that is, adherence to screening for future pregnancy intention and specific reasons for short-term ICC, as well as what action was taken per six-months consultation in which ICC was discussed); *Adoption*, defined as the uptake of discussing ICC measured among PCHC professionals; *Feasibility*, referred to as the expected possibility of ICC integration in PCHC among professionals; *Appropriateness*, being the desirability of ICC in PCHC among professionals and women; and *Acceptability*, that is, the agreeability on aspects of ICC in PCHC among professionals and women [20, 29]. Lastly, the *effectiveness* of the intervention was studied as the uptake (i.e. the number) of separate ICC consultations.

### Data collection

Data were collected at three levels (Fig 1): data from records kept at each PCHC well-baby clinic, questionnaires filled out by PCHC professionals, and questionnaires filled out by participating women who considered becoming pregnant. From the different ways of data collection that were used, all items on the implementation outcomes are outlined in detail in S1 Table.

PCHC records were used to collect data on coverage: the total number of six-months consultations and whether during these consultations ICC was discussed. In addition, if women gave consent, we extracted from the PCHC records also data about specific findings during this discussion (i.e. pregnancy intention and actions taken; referred to as fidelity) and certain background characteristics (i.e. age, ethnicity, parity, and 4-digit postal code to determine neighborhood deprivation ‘yes’/’no’ as previously defined [30]). The uptake of ICC consultations was also registered through PCHC records. The data from PCHC records was either extracted from PCHC electronic records or took complementary place on paper (i.e. in case integration of data collection of ICC items was not possible in the electronic records). It was then anonymized and transferred into a Generic Medical Survey Tracking system called Gemstracker (https://gemstracker.org/general-information).

The content of the questionnaire for PCHC professionals was comparable for both subgroups of PCHC teams participating and not-participating in the intervention, however it included more detailed questions on the intervention for the participating teams. It contained data on participation in ICC (i.e. adoption), determinants of implementation as developed in previous studies (i.e. feasibility, appropriateness, and acceptability)[31], and background characteristics (e.g. age, work experience).

The professionals participating in the intervention were requested to respond to the digital questionnaire twice: once three months into the intervention and again at the end of the intervention period. Members of non-participating PCHC teams were requested to respond once to the digital questionnaire.

All women who considered becoming pregnant and agreed to participate in the questionnaire study were asked for their email address and were subsequently sent a digital questionnaire. It contained questions about background characteristics (i.e. age, ethnicity, educational attainment, number of children, income, civil status), medical and obstetrical history, lifestyle behaviors, and women’s opinion regarding appropriateness and acceptability of ICC. A second questionnaire was sent six months later to assess uptake and effectiveness of ICC.

The intervention and data collection started in alignment with preferences of each municipality. The first municipality started data collection in December 2015; the last municipality started in September 2016. The intervention lasted up to and including February 2017.

### Data analyses

Descriptive statistics were performed to describe background characteristics of the municipal PCHC services, the PCHC professionals participating in the questionnaire study and the participating women. Frequencies and percentages were used to describe the implementation outcomes. In describing the coverage, minimum and maximum values over the different municipalities were also analyzed, as well as the results per ICC delivery approach (i.e. PCHC or GPs and midwives; Fig 1). With respect to acceptability by PCHC professionals, a composite outcome based on the eight different questionnaire items (S1 Table) was used and both the median score and the percentage of professionals was determined based on the composite score agreed with the items (i.e. average ≥3.5; range 1–5). For the composite outcome the Cronbach's alpha was calculated to assess the internal consistency of items. Data analyses were performed with SPSS Statistics (version 21).

### Ethics approval and consent to participate

The study was reviewed by the Daily Board of the Medical Ethics Committee Erasmus MC in the Netherlands (MEC-2015-182). As a result of this review, the Board declared that this research proposal is not obliged to apply the rules laid down in the Medical Research Involving Human Subjects Act. Written informed consent was obtained from the women who participated in the questionnaire study. We obtained consent from the PCHC organizations to send their professionals a request to participate in our digital questionnaire study. The PCHC professionals who were eligible to participate in the questionnaire study could opt out from participation.

## Results

### Organizational level

#### Organizational characteristics

**Fig 2:**
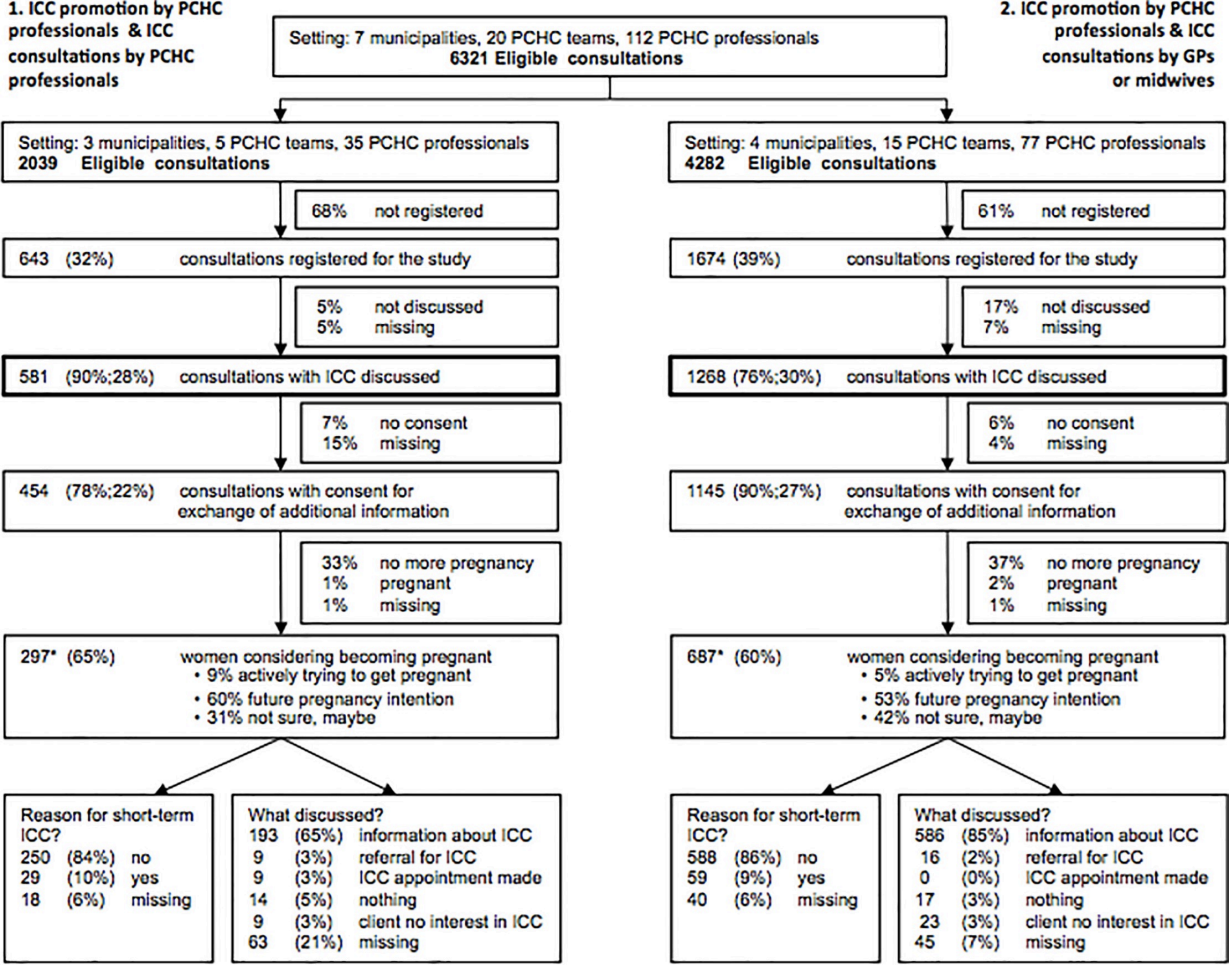
Overview of ICC implementation (coverage and fidelity) by ICC delivery approach. (%; %): first % refers to the total number in de line above, the second % refers to the absolute total number of consultations. *Total number of women considering a pregnancy is 984 (297+687).

The intervention period ranged from six to 13 months per municipality. A total of 21 teams were trained at the beginning of the study and a total of 20 PCHC teams participated in the intervention throughout the study (Fig 2), ranging from one to ten per municipality. One trained PCHC-team did not start the intervention due to being understaffed because of sick leave. The number of PCHC professionals involved was 112 and varied per municipality from three to 28. In total, 6,321 six-months consultations took place during the study period (ranging from 192 to 1,726 per municipality).

#### Coverage, fidelity, and effectiveness

ICC was discussed in 1,849 consultations and as such the coverage of our intervention was 29% of the total amount of 6,321 six-months consultations. The coverage did not differ per delivery approach (Fig 2), but did vary between 12% and 55% per municipality. Additional characteristics from the PCHC records were available for 86% (n = 1,599) of the women with whom ICC was discussed; Of these women with whom ICC was discussed 62% (n = 984) considered becoming pregnant again. Of the 984 women considering to become pregnant, the median age was 30 years (min-max: 16–43 years), 32% did not consider themselves of Dutch background, 19% lived in a deprived neighborhood, and 40% were multiparous.

In addition, PCHC professionals identified reasons for short-term ICC, which meant either already trying to get pregnant or having an obstetrical history of an adverse perinatal outcome, in ten percent of these 984 women. Professionals’ actions consisted of information provision about ICC in 80% of the 984 women. Out of the seven participating municipalities, only one was successful in realizing any separate ICC appointments. In this municipality some PCHC professionals not only provided information, but also proactively arranged the ICC appointments. Nine separate ICC appointments were made, of which four actually took place.

### PCHC professionals

#### Characteristics of the professionals

Of the total number of participating PCHC professionals (n = 112), 70% (n = 78) responded to the first questionnaire (Q1). At the time of the second questionnaire (Q2), 99 (88%) professionals were still working in the participating teams and 66% (n = 65) of these professionals responded. Professionals from all seven municipalities were represented in the responses to both questionnaires. The questionnaire to non-participating teams was sent to 394 professionals, of which 116 (29%) responded. After excluding professionals who reported awareness of the HP4All program, 91 (78%) questionnaires were available.

Baseline characteristics of the PCHC professionals who responded to the questionnaires are presented in Table 1. Relatively more PCHC nurses than physicians replied to the questionnaire in the non-participating teams (74%) than in the participating teams (54%).

**Table 1:** Characteristics and implementation outcomes of PCHC professionals in participating and non-participating teams.

Characteristics and implementation outcomes of PCHC professionals	Participating team Q1 N = 78		Participating team Q2 N = 65		Non-participating team N = 91	
**Age (years)**	45.0	22–66	46.0	22–66	44.0	21–64
**Profession**						
** physician**	36	46.2%	30	46.2%	24	26.4%
** nurse**	42	53.8%	35	53.9%	67	73.6%
**Work experience in current function (years)**	9.0	1–37	10.0	1–35	9.0	0–35
**Received training about ICC (yes)**	62	79.5%	NA	NA	3	3.3%
**How well-informed about ICC (well)**	NA	NA	41	63.1%	4	4.4%
***Adoption*: Attention to promotion or delivery of ICC (not little / not much—much—very much)**	NA	NA	36	56.3%	14	15.4%
***Adoption*: Asking about intention to become pregnant (≥ 50% women)**	31	41.3%	25	39.1%	7	7.7%
***Adoption*: Informing clients about ICC in case of known future pregnancy intention (≥ 50% women)**	30	39.5%	30	46.9%	3	3.3%
*Feasibility*: ICC in PCHC probable (yes)*	21	27.3%	8	12.5%	11	12.1%
*Appropriateness*: ICC in PCHC desirable (yes)*	35	44.9%	30	46.9%	41	45.1%
*Acceptability*: Important to contribute to ICC (agree)**	48	61.5%	31	53.4%^**1**^	48	53.3%
*Acceptability*: Composite statement outcome (agree)**	31	39.7%	21	36.2%^**1**^	33	36.7%
***Acceptability*: Composite statement outcome (median)** ***	3.38	2.5–5.0	3.31	2.4–4.8^**1**^	3.25	2.0–4.4

Median with min–max or numbers with percentages of non-missing cases. Missing value <5% unless otherwise stated. NA: Not available.

* Instead of 'maybe' or 'no'.

** Instead of neutral ' or 'disagree'

*** Possible scores ranged from 1–5.

^1^ Missings > 5% (10.8%)

#### Adoption, feasibility, appropriateness, and acceptability

The implementation outcomes based on the three questionnaires among PCHC professionals are presented in Table 1. At the end of the study period (Q2), adoption of regularly informing clients about ICC was 46.9% among all PCHC professionals. This was higher among the 30 physicians (60.0%), who usually provide the six-months PCHC consultation. These physicians selected the following reasons for not discussing ICC most often: ‘not enough time due to other tasks’ (63.3%), ‘difficult communication’ (50%), and 'I forgot' (46.7%). With regards to possible suggested forms of ICC, the physicians agreed with the following forms of ICC most often: 'providing information materials' (83.3%), 'discussing referral for ICC at GPs or midwives' (67.7%), 'providing general advice during routine PCHC visits' (60.0%), and 'screening for risk factors and discussing these during routine visits' (46.7%). They agreed least often with 'Performing an actual ICC consultation' (23.3%).

Feasibility, appropriateness, and acceptability were similar in participating and non-participating PCHC teams (Table 1). Feasibility was considerably lower than appropriateness and acceptability (Table 1). In all groups, the majority was unsure about the feasibility (range 68.8–79.1%) and 3.9–11.0% expected integration of ICC in PHCH not to be feasible. The reported explanations for expected low feasibility were 'not enough resources' (i.e. time and financial compensation) and 'dependence on prioritizations of the PCHC organization and municipality', while 'sufficient training' was mentioned as a requirement. With regards to appropriateness, some professionals were unsure and mentioned that ICC 'does not fit in the current tasks of PCHC' and 'might be more suitable for GPs and midwives', and that they 'expected little interest from the target group'. However, most explanations for appropriateness were along the lines that ICC in PCHC is 'relevant' (i.e. importance of prevention, reproductive planning, and reaching vulnerable groups) and 'suitable' within the preventive tasks and reach of PCHC. Regarding acceptability, very few professionals disagreed with the statement that ‘it is important to contribute to ICC’ (Q1: 1.3%, Q2: 1.7%, and non-participating teams: 7.8%).

### Women participating in the questionnaire study

#### Characteristics of the participants

**Fig 3:**
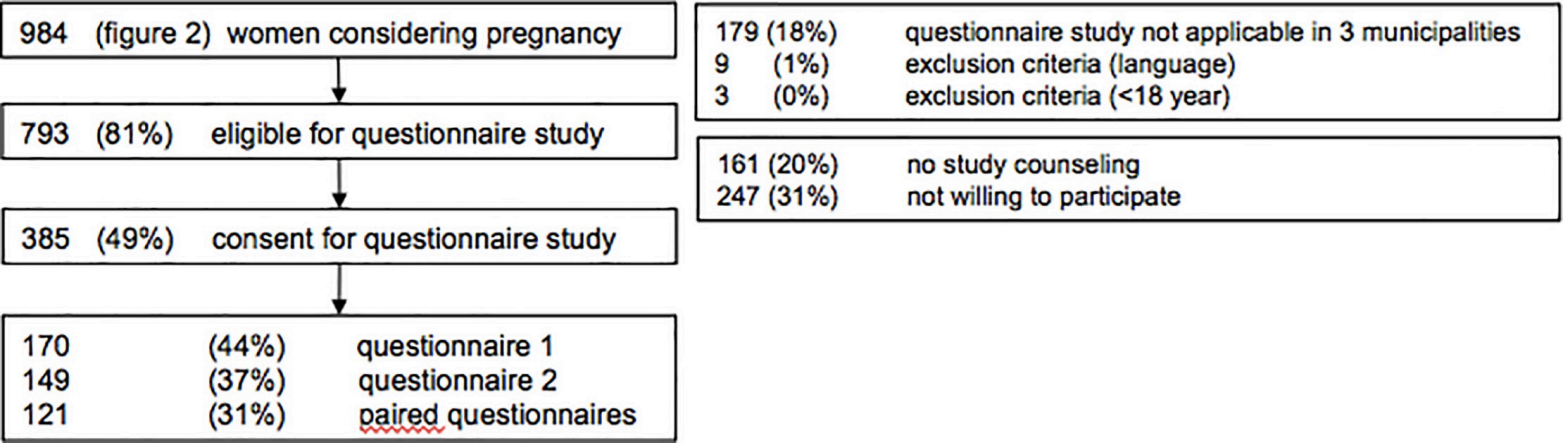
Flowchart of participants (women) in the study.

Of the 984 women who considered a future pregnancy (Fig 2*), 793 women were eligible to participate in our questionnaire study (Fig 3). In total, 385 women (49%) consented to participate in the questionnaire study, of whom 170 (44%) responded to the first questionnaire and 149 (37%) responded to the second questionnaire. Baseline characteristics of the participants are displayed in S2 Table. It shows the prevalence of potential interconceptional risk factors for adverse pregnancy outcomes, such as a complicated obstetric history (23.7%) and no preconceptional folic acid supplementation before a previous pregnancy (31.1%).

#### Appropriateness, acceptability, and effectiveness

Certain items from the questionnaires had missing data, therefore, frequencies and percentages represent the number of available responses. In questionnaire 1, with regards to appropriateness, most women (n = 129, 94.2%) agreed to the statement “I should receive information about ICC via well-baby clinics”. With respect to acceptability, the majority (n = 93, 66.4%) also agreed to the statement: “I find it acceptable that I was asked whether I consider becoming pregnant again”, whereas 4.2% disagreed and 29.4% was neutral.

In the second (follow-up) questionnaire, only one woman reported to have had an ICC consultation (effectiveness). To the question whether women considered making use of an ICC consultation in the future, two women (1.4%) replied “Yes”, 55 women (38.7%) “Maybe”, and 85 women (59.9%) “No”. When participants were asked about their reasons for not planning an ICC consultation, the following reasons were reported (n = 70): 55.7% “was not convinced about the benefit”; 31.4% “did not know what it would entail”; 8.6% “was unable to go to an appointment”; and in 4.3% the “partner did not consider it to be necessary”.

## Discussion

### Principal findings

This study has shown certain possibilities to promote ICC in PCHC, and at the same time it has illustrated important challenges regarding promotion and actual delivery of ICC. After introducing the intervention, PCHC physicians discussed the possibility of an ICC consultation with mothers in about a third of the routine PCHC visits at the child's age of six months. Promising is that the majority of PCHC physicians adopted the promotion of ICC and that many professionals judged integrating ICC in PCHC as appropriate and acceptable. However, even in the best performing municipality coverage did not exceed 55%, showing limited implementation. Possibly, either the urgency of promoting ICC was not conveyed enough to the PCHC professionals or feasibility concerns related to lack of time formed insurmountable barriers for successful implementation. This shows, together with low uptake of ICC among women, the challenge of delivering ICC. Although women were positive with regards to being informed about ICC, they could not be convinced to make an appointment for an ICC consultation.

The field of implementation research is increasingly acknowledged in its attempt to optimize the translation of evidence-based insights into practice [20, 32]. Implementation research may provide valuable insights with regards to PCC and ICC, since daily practice is still uncommon. One Spanish study based on implementation outcomes has recently suggested that the possibility of integrating a simple general preventive screening intervention for healthy lifestyles in primary care is promising [33]. This study showed higher overall coverage (52%) and adoption rates (75%) than our study [33]. More specifically for PCC and ICC, a few studies have already shown that acceptability of pregnancy intention screening in primary care is high [34, 35]. As such, screening pregnancy intention in primary care has been advocated as a strategy to promote both preconception care as well as contraceptive care for women [36]. However, with regards to the effectiveness of such screening on uptake of care, little remains known [34].

In our study, uptake of ICC was low as only few women had an appointment for an ICC consultation. Appointments only occurred in one municipality where the PCHC professionals pro-actively arranged it. Women themselves did not seem to make ICC appointments and they reported a low need and unfamiliarity with ICC as barriers for making an appointment. These barriers for uptake of ICC among women have been recognized as important barriers before [37]. Even though the aim of our intervention was to overcome these barriers by promotion of ICC by PCHC professionals, it appeared insufficient to substantially improve the uptake of ICC. It could be that PCC and ICC are still too unfamiliar. Women should encourage each other, they should be empowered to seek information about preparing for pregnancy and get confirmation from their peers that visiting a healthcare professional for PCC of ICC is a sensible thing to do. A suitable medium for this could be Centering Parenting [38].

Possibly, ICC could also become more common by further integration of general ICC health promotion within routine care provided by PCHC teams. It would diminish the currently found barrier among women of organizing a separate appointment and could also reduce the barriers among professionals when this routine care would be mentioned in a national guideline and would be sufficiently compensated. Furthermore, the acceptability among both groups seemed to be good with regards to integration of ICC topics in routine care. While a separate ICC consultation with other professionals such as GPs, midwives, or gynecologists could still be an opportunity in case of detected higher risk for adverse pregnancy outcomes, awareness of certain ICC topics among professionals and women would at least be secured. For instance, other studies focusing on the promotion of folic acid supplementation in routine PCHC practice have shown promising results with regards to increased use and intention [18, 19]. Other encouraging, recently reported, ICC related practices that were aimed at mothers during well-child visits, include screening and addressing tobacco use, depression risk and contraception use [39, 40]. As such, standardization of certain ICC items in PCHC could make it accessible for all women while warranting sufficient management support and resources, which could improve feasibility. PHCH professionals judged feasibility problematic due to for instance limited resources (e.g. time) and too little prioritization by their organizations. This is in line with the expected barriers based on the previously published study on determinants for implementation of ICC in PCHC [28]. Together, above-mentioned studies call for a national guideline on ICC in PCHC.

### Strengths and limitations

Strengths of this study are introducing the ICC intervention in the real-time practice of PCHC, including training of professionals, and evaluating this intervention in a comprehensive way. We included data from different sources, representing different stakeholders, which contributed to such comprehensive evaluation, as has been suggested for implementation research [20, 29, 32]. Our study also has some limitations. Firstly, the implementation outcomes costs and sustainability were not included in our study. Secondly, we only measured limited effectiveness of our intervention on uptake of ICC and we could not measure the effectiveness on health outcomes. Thirdly, a selection bias may have occurred in participating professionals and women with regards to their opinion on ICC, since participation rates in some of the questionnaires were rather low. Also, registration in the PCHC records seemed often only performed in case ICC was discussed and hence certain study outcomes were only available in 37% of the total six-months consultations. Lastly, municipal differences in for instance management involvement, time constraints, staffing issues, and other context factors such as restructuring PCHC, likely influenced differences between municipalities, but separate analyses on these factors were outside the scope of this study.

## Conclusion

In 29% of the routine PCHC visits, a separate ICC consultation was promoted, however this is insufficient to reach women with the provision of ICC. Suggestions for improvement include further integration of ICC health promotion in routine PCHC consultations, while allocating sufficient resources (e.g. time, financial compensation, training, and a national guideline) to increase feasibility. These possibilities are worthwhile to further investigate, given the unique opportunity of PCHC services to access women of reproductive age with preventive ICC.

## Supporting information

S1 Table Outline of implementation outcomes as derived from the questionnaires. (DOCX)

S2 Table Characteristics of participants. (DOCX)

S1 Questionnaire Questionnaire Professionals 1. (PDF)

S2 Questionnaire Questionnaire Professionals 1 NL. (PDF)

S3 Questionnaire Questionnaire Professionals 2. (PDF)

S4 Questionnaire Questionnaire Professionals 2 NL. (PDF)

S5 Questionnaire Questionnaire Women1. (PDF)

S6 Questionnaire Questionnaire Women1 NL. (PDF)

S7 Questionnaire Questionnaire Women2. (PDF)

S8 Questionnaire Questionnaire Women2 NL. (PDF)
