# Supplementary material for: Integrating interconception care in preventive child health care services: The Healthy Pregnancy 4 All program
Source: PLoS One. 2019 Nov 6;14(11):e0224427. doi: 10.1371/journal.pone.0224427 (PMC6834275; doi:10.1371/journal.pone.0224427)
Supplement: S1 Table — (DOCX) [file pone.0224427.s001.docx]

**S1 Table**. Outline of implementation outcomes as derived from the questionnaires

| Outcomes | Data collection /source | Items | Response categories | Scoring / reporting |
| --- | --- | --- | --- | --- |
| Coverage | PCHC records | Was the possibility of ICC discussed? | Yes – no – unknown/missing | % yes |
| Fidelity | PCHC records | Screening for intention to become pregnant? | Actively trying to become pregnant – future pregnancy intention – not sure yet, maybe – currently pregnant – no more pregnancy intention – unknown/missing | % response to answer categories |
|  |  | In case of possible pregnancy intention; reasons for short-term ICC (e.g. actively trying to become pregnant or obstetrical history of adverse pregnancy outcome) | Yes – no – unknown/missing | % response to answer categories |
|  |  | In case of possible pregnancy intention, what action was taken during the six-month consultation? | Just information provision about ICC – referral for ICC – ICC appointment made – nothing – client had no interest – unknown/missing | % response to answer categories |
| Adoption | PCHC provider questionnaires (2/3)^a^ | How much attention do you pay to providing ICC? | very little - little - not little / not much - much - very much | not little / not much - much - very much |
|  | PCHC provider questionnaires | With how many women do you discuss whether she intends/considers to become pregnant again? | none - a minority - half - a majority - everyone | ≥ 50% women |
|  |  | With how many women do you discuss the possibility of an ICC consultation? | none - a minority - half - a majority - everyone | ≥ 50% women |
|  | PCHC provider questionnaires (1/3)^b^ | In case you are not able to discuss ICC, what was the main reason? | \| Lack of time due to my other tasks \| \| --- \| \| Lack of time due to late arrival of the client \| \| I experience not enough expertise \| \| I do not consider it my task \| \| It feels not right due to circumstances of the client \| \| The client does not want to discuss it \| \| Difficult communication (e.g. language barrier \| \| I forgot \| \| Other reason \| | Multiple answers possible, reported if >33% responses |
| Feasibility | PCHC provider questionnaires | Do you expect that ICC will actually be integrated in PCHC in the future? | very certainly not - certainly not - maybe not/yes -  certainly yes - very certainly yes | certainly yes - very certainly yes |
|  |  | Explanation |  | Summary of responses |
| Appropriateness | PCHC provider questionnaires | Do you consider it desirable that ICC will actually be integrated in PCHC in the future? | very certainly not - certainly not - maybe not/yes -  certainly yes - very certainly yes. | certainly yes - very certainly yes |
|  |  | Explain |  | Summary of responses |
|  |  | If ICC becomes integrated in PCHC, do you find these forms appropriate?^b^  - providing information materials  - providing general advice during routine PCHC visits  - screening for risk factors and discussing these during routine visits  - performing an actual ICC consultation  - discussing referral for ICC at GPs or midwives | very certainly not - certainly not - maybe not/yes -  certainly yes - very certainly yes. | certainly yes - very certainly yes. |
|  | Participant Q1 | How do you think that you should receive information about the existence of an ICC consultation? | “PCHC - well-baby” clinic could be selected | yes |
| Acceptability | PCHC provider questionnaires | ICC is as far as I know based on empirical evidence | strongly disagree - disagree - neutral - agree - strongly agree | agree - strongly agree |
|  |  | ICC is in line with how I am used to work | strongly disagree - disagree - neutral - agree - strongly agree | agree - strongly agree |
|  |  | I think it is important to contribute to ICC | strongly disagree - disagree - neutral - agree - strongly agree | agree - strongly agree |
|  |  | I think it is my job to provide ICC | strongly disagree - disagree - neutral - agree - strongly agree | agree - strongly agree |
|  |  | I have sufficient knowledge and skills to be able to provide ICC | strongly disagree - disagree - neutral - agree - strongly agree | agree - strongly agree |
|  |  | I find interconception care suitable for my clients | strongly disagree - disagree - neutral - agree - strongly agree | agree - strongly agree |
|  |  | I expect that clients will generally be satisfied if I provide ICC | strongly disagree - disagree - neutral - agree - strongly agree | agree - strongly agree |
|  |  | I expect that clients will generally cooperate if I provide ICC | strongly disagree - disagree - neutral - agree - strongly agree | agree - strongly agree |
|  | Participant Q1 | I think that it is good that I was asked whether I consider becoming pregnant again | strongly disagree - disagree - neutral - agree - strongly agree | agree - strongly agree |
| Effectiveness | PCHC records and records from GP and midwifery practices | Registration of ICC consultations |  | Total number |
|  | Participant Q2 | Did you have an appointment for an ICC consultation? | yes - no | % response to answer categories |
|  |  | Do you intend to have an ICC consultation in the future? | yes- maybe - no | % response to answer categories |
|  |  | What was the most important reason for you to decide not to have an ICC consultation? | I dreaded having an appointment - I was not convinced about the benefit - I did not know what it would entail - I was unable to go to an appointment - I could not get an appointment with the healthcare provider that I wanted to visit - my partner did not consider it necessary | % response to answer categories |
| Questionnaires:  Participant Q1 + Q2. PCHC providers: Participating team Q1 + Q2 and Non-participating team Q1.  a. not participating team Q1  b. only participating team Q2 | | |  | |
