# Supplementary material for: Integrating interconception care in preventive child health care services: The Healthy Pregnancy 4 All program
Source: PLoS One. 2019 Nov 6;14(11):e0224427. doi: 10.1371/journal.pone.0224427 (PMC6834275; doi:10.1371/journal.pone.0224427)
Supplement: S2 Table — (DOCX) [file pone.0224427.s002.docx]

**S2 Table.** Characteristics of participants

| **Characteristics at baseline (Q1) N= 170** ^a^ | | **N** | **%** |
| --- | --- | --- | --- |
| Age | Median age in years (min- max) | 30.5 | 20-43 |
| Ethnicity ^b^ | Dutch | 133 | 85.3 |
|  | Other | 23 | 14.7 |
|  | Missing | 14 | 8.2 |
| Educational attainment ^c^ | Low | 6 | 3.9 |
|  | Intermediate | 47 | 30.3 |
|  | High | 102 | 65.8 |
|  | Missing | 15 | 8.8 |
| Pregnancy intention | Currently pregnant | 1 | 0.7 |
|  | Within next 6 months | 14 | 9.3 |
|  | Within next 6 - 12 months | 23 | 15.2 |
|  | After > 12 months | 78 | 51.6 |
|  | In doubt about becoming pregnant again | 35 | 23.2 |
|  | Missing | 19 | 11.2 |
| How many living children | One child | 124 | 81 |
|  | Missing | 17 | 10 |
| Paid job | Yes | 136 | 87.7 |
|  | No | 19 | 12.3 |
|  | Missing | 15 | 8.8 |
| Monthly household income | Low (<1500€) | 7 | 4.1 |
|  | Middle (1500 - 3000€) | 55 | 32.4 |
|  | High (>3000€) | 91 | 53.5 |
|  | Missing | 17 | 10.0 |
| Civil status | Living together | 148 | 96.1 |
|  | In a relationship, not living together | 4 | 2.6 |
|  | Not in a relationship | 2 | 1.3 |
|  | Missing | 16 | 9.4 |
| Obstetric history | Low birth weight baby (<2500gram) | 13 | 9.4 |
|  | Child with congenital abnormalities | 4 | 2.9 |
|  | Preterm birth (<37 weeks) | 16 | 11.5 |
|  | Medical concerns of the neonate following birth | 15 | 10.9 |
|  | Perinatal mortality | 3 | 2.2 |
|  | Composite outcomes (1 of 5 outcomes above) | 33 | 23.7 |
|  | Missing | 33 | 19.4 |
| Diabetes, hypertension or | Yes | 15 | 10.7 |
| pre-eclampsia | No | 125 | 89.3 |
|  | Missing | 30 | 17.6 |
| Preconception lifestyle risks | No folic acid supplementation | 132 | 86.8 |
|  | No folic acid before last pregnancy | 46 | 31.1 |
|  | Smoking | 15 | 9.8 |
|  | Alcohol consumption ≥ 1/week | 104 | 68 |
|  | Illicit drug use | 1 | 0.7 |
|  | Missing | 18 | 10.6 |
| Chronic medical condition | Yes | 15 | 10.1 |
|  | No | 134 | 89.9 |
|  | Missing | 21 | 12.3 |
| Contraception | Yes | 115 | 76.2 |
|  | No | 36 | 23.8 |
|  | Missing | 19 | 11.2 |
| 1. Data are expressed as numbers and percentages of non-missing cases unless otherwise specified. Missing value percentage of total. 2. Self-defined ethnicity 3. Educational attainment level was defined as the highest completed educational level classified according to the International Standard Classification of Education (ISCED) i.e. low (level 0-2: early childhood; primary education; lower secondary education); intermediate (level 3-5: upper secondary; post-secondary; short cycle tertiary); and high (level 6-8: bachelor; master; doctoral). Unesco institute for statistics 2014. | | | |
