# Supplementary material for: Integrating interconception care in preventive child health care services: The Healthy Pregnancy 4 All program
Source: PLoS One. 2019 Nov 6;14(11):e0224427. doi: 10.1371/journal.pone.0224427 (PMC6834275; doi:10.1371/journal.pone.0224427)
Supplement: S1 Questionnaire — (PDF) [file pone.0224427.s003.pdf]

Interconception care HP4All-2 Questionnaire

Preventive Child Health Care professionals

Baseline characteristics

What is your job?

In which municipality do you work?

In which team or at which location (s) do you work?

What is your age?

How many years of work experience do you have?

How many years of work experience do you have in your current position?

Current situation:

Did you attend the training on interconception care?

- no
- yes

Which tasks with regard to the implementation of interconception care are expected of you?

- none
- Asking about intention to become pregnant (child wish)
- provide materials with information
- provide general information and advice
- inform about the possibility of a pre-pregnancy consultation
- refer to a separate appointment for a pre-pregnancy consultation (in case of a child wish)
- carrying out a pre-pregnancy consultation (in the case of a child wish)
- I do not know

To what extent do you perform interconception care tasks now?

With how many clients?

	none	A minority	half	A majority	every one
**Asking about intention to become pregnant (child wish)**					
**Providing materials with information**					
**Providing general information and advice**					
**Informing about the possibility of a pre-pregnancy consultation**					
**Referring to a separate appointment for a pre-pregnancy consultation (in case of a child wish)**					
**Carrying out a pre-pregnancy consultation (in the case of a child wish)**					

If you are unable to perform interconception care tasks, can you indicate why that is mainly due to this?

- Lack of time due to my other tasks
- Lack of time due to late arrival of the client
- I experience insufficient expertise
- I do not consider it my task
- It feels not right due to circumstances of the client
- The client does not want to discuss it
- Difficult communication with the client (such as a language barrier or low health skills)
- I forgot
- Other reason

Assuming that the consultation has taken place.

This question is about your opinion and your expectation with regard to interconception care within PCHC

	Very certainly not	Certainly not	maybe	Certainly yes	Very certainly yes
To what extent do you consider it desirable that PCHC will provide interconception care now and in the future?					
To what extent do you expect PCHC to provide interconception care now and in the future?					

Statements

	Strongly disagree	Dis-agree	neutral	agree	Strongly agree
As far as I know, interconception care is based on empirical evidence					
Interconception care is too complicated for me to provide					
ICC is in line with how I am used to work					
I think it is important to contribute to ICC					
I think it is my job to provide ICC					
I have sufficient knowledge and skills to be able to provide ICC					
By providing interconception care I can develop more substantively					
By providing interconception care I can develop less substantively					
I find interconception care suitable for my clients					
I expect that clients will generally be satisfied if I provide ICC					
I expect that clients will generally cooperate if I provide ICC					

	Very certainly not	Certainly not	maybe	Certainly yes	Very certainly yes
**I expect that by providing interconception care, I will make my clients more consciously prepare for a new pregnancy**					

Space for comments

Do you wish to comment on this questionnaire or on interconception care in general?

Thank you for participating in this survey.
