# Supplementary material for: Integrating interconception care in preventive child health care services: The Healthy Pregnancy 4 All program
Source: PLoS One. 2019 Nov 6;14(11):e0224427. doi: 10.1371/journal.pone.0224427 (PMC6834275; doi:10.1371/journal.pone.0224427)
Supplement: S2 Questionnaire — (PDF) [file pone.0224427.s004.pdf]

Vragenlijst
Zorgverleners Interconceptiezorg HP4All-2

Basiskarakteristieken

Wat is uw functie? *

In welke gemeente werkt u? *

In welk team of op welke locatie(s) werkt u?

Vul uw antwoord hier in:

Wat is uw leeftijd?

Hoeveel jaren werkervaring heeft u?

Hoeveel jaren werkervaring heeft u in uw huidige functie?

De volgende twee vragen gaan over uw werk voordat het HP4All-2 interconceptiezorg project was gestart.

Bij hoeveel clienten voerde u al interconceptiezorg uit in onderstaande vormen?

Kies het toepasselijke antwoord voor elk onderdeel:

	geen enkele	een minderheid	de helft	een meerderheid	iedereen
**Bespreken van een kinderwens**					
**Materialen meegeven met informatie**					
**Algemene inhoudelijke informatie en adviezen geven**					
**Informeren over de mogelijkheid van een kinderwensconsult**					
**Verwijzen naar een aparte afspraak voor een kinderwensconsult (bij een kinderwens)**					
**Verrichten van een kinderwensconsult (bij een kinderwens)**					

Huidige situatie

Heeft u de nascholing over interconceptiezorg bijgewoond?*

Kies één van de volgende mogelijkheden:

- nee
- ja

Welke taken ten aanzien van de uitvoering van interconceptiezorg worden van u verwacht?

- geen
- bespreken van een kinderwens
- materialen meegeven met informatie
- algemene inhoudelijke informatie en adviezen geven
- informeren over de mogelijkheid van een kinderwensconsult
- verwijzen naar een aparte afspraak voor een kinderwensconsult (bij een kinderwens)
- het verrichten van een kinderwensconsult (bij een kinderwens)
- dat weet ik niet

In hoeverre voert u interconceptiezorg taken nu uit? Bij hoeveel clienten?

Kies het toepasselijke antwoord voor elk onderdeel:

	geen enkele	een minderheid	de helft	een meerderheid	iedereen
**Bespreken van een kinderwens**					
**Materialen meegeven met informatie**					
**Algemene inhoudelijke informatie en adviezen geven**					
**Informeren over de mogelijkheid van een kinderwensconsult**					
**Verwijzen naar een aparte afspraak voor een kinderwensconsult (bij een kinderwens)**					
**Verrichten van een kinderwensconsult (bij een kinderwens)**					

Indien het u niet lukt om interconceptiezorg taken uit te voeren, kunt u dan aangeven waardoor dat voornamelijk komt?

Selecteer alle mogelijkheden:

- geen tijd vanwege mijn andere taken
- geen tijd omdat de client te laat komt
- ik ervaar onvoldoende expertise
- ik vind het niet mijn taak
- het voelt niet goed vanwege de persoonlijke situatie van de client
- de cliënt staat er niet voor open
- moeizame communicatie met de client (zoals een taalbarriere of lage gezondheidsvaardigheden)
- ik ben het vergeten om uit te voeren

Ervan uitgaande dat het consult wel heeft plaatsgevonden.

Deze vraag gaat over uw mening en uw verwachting ten aanzien van interconceptiezorg binnen de JGZ

Kies het toepasselijke antwoord voor elk onderdeel:

	zeer zeker niet	zeker niet	misschien niet, misschien wel	zeker wel	zeer zeker wel
**In hoeverre acht u het wenselijk dat de JGZ nu en in de toekomst interconceptiezorg zal gaan geven?**					
**In hoeverre verwacht u dat de JGZ nu en in de toekomst interconceptiezorg zal gaan geven?**					

Stellingen

Kies het toepasselijke antwoord voor elk onderdeel:

	helemaal mee oneens	mee oneens	noch mee oneens, noch mee eens	mee eens	helemaal mee eens
**Interconceptiezorg is voor zover ik weet, gebaseerd op wetenschappelijke kennis**					
**Interconceptiezorg is te ingewikkeld voor mij om te geven**					
**Interconceptiezorg sluit aan bij hoe ik gewend ben om te werken**					
**Ik vind het belangrijk om een bijdrage te leveren aan interconceptiezorg**					
**Ik vind het tot mijn functie behoren om interconceptiezorg te geven**					
**Ik beschik over voldoende kennis en vaardigheden om interconceptiezorg te kunnen geven**					
**Ik kan mij door het geven van interconceptiezorg meer inhoudelijk ontwikkelen**					
**Ik kan mij door het geven van interconceptiezorg minder inhoudelijk ontwikkelen**					
**Ik vind interconceptiezorg geschikt voor mijn clienten**					

Ik verwacht dat cliënten over het algemeen tevreden zullen zijn als ik interconceptiezorg geef

Ik verwacht dat cliënten over het algemeen zullen meewerken als ik interconceptiezorg geef

Kies het toepasselijke antwoord voor elk onderdeel:

	zeer zeker	zeker niet	misschien niet, misschien wel	zeker wel	zeer zeker wel
**Ik verwacht dat ik door het geven van interconceptiezorg bereik dat mijn cliënten zich bewuster voorbereiden op een nieuwe zwangerschap**					

Ruimte voor opmerkingen

Vul uw antwoord hier in:

Wilt u naar aanleiding van deze vragenlijst of naar aanleiding van interconceptiezorg in het algemeen, nog aanvullende opmerkingen plaatsen? Maakt u dan gebruik van bovenstaande ruimte.

Bedankt voor uw deelname aan deze enquête.
