# Supplementary material for: Integrating interconception care in preventive child health care services: The Healthy Pregnancy 4 All program
Source: PLoS One. 2019 Nov 6;14(11):e0224427. doi: 10.1371/journal.pone.0224427 (PMC6834275; doi:10.1371/journal.pone.0224427)
Supplement: S3 Questionnaire — (PDF) [file pone.0224427.s005.pdf]

Interconception care HP4All-2 Questionnaire 2

Preventive Child Health Care professionals

Baseline characteristics

What is your age?

How many years of work experience do you have in your current position?

Interconception care – awareness

To what extent do you think you are aware of what interconception care entails?

- Before reading the introduction, I had no idea what interconception care meant
- I had heard of it before, but I cannot say what it means
- I know broadly what interconception care means, but I do not know any substantive details
- I am reasonably informed and know substantive details
- I am well aware of the content and could explain this to someone

Current situation

The following questions are about the extent to which attention is paid at your workplace to the provision of interconception care. This includes flyers about healthy pregnancy, training courses offered on this subject, conversations with colleagues about providing care to women with a desire to have children, and so on.

	Very little	little	Not little, not much	much	Very much
**How much attention is paid to providing ICC at your PCHC location?**					
**How much attention do you think your colleagues pay to providing ICC?**					
**How much attention do you pay to providing ICC?**					

To what extent do you perform interconception care tasks now?

With how many clients?

	none	A minority	half	A majority	every one
**Asking about intention to become pregnant (child wish)**					
**Providing materials with information**					
**Providing general information and advice**					
**Informing about the possibility of a pre-pregnancy consultation**					
**Referring to a separate appointment for a pre-pregnancy consultation (in case of a child wish)**					
**Carrying out a pre-pregnancy consultation (in the case of a child wish)**					

If you are unable to perform interconception care tasks, can you indicate why that is mainly due to this?

- Lack of time due to my other tasks
- Lack of time due to late arrival of the client
- I experience insufficient expertise
- I do not consider it my task
- It feels not right due to circumstances of the client
- The client does not want to discuss it
- Difficult communication with the client (such as a language barrier or low health skills)
- I forgot
- Other reason

Assuming that the consultation has taken place.

Interconception care – future

The following questions are about your opinion regarding interconception care within PCHC

To what extent do you consider it desirable that PCHC will provide interconception care now and in the future?

- **Very certainly not**
- **Certainly not**
- **Maybe yes, maybe not**
- **Certainly yes**
- **Very certainly yes**

Could you explain your answer?

To what extent do you expect PCHC to provide interconception care now and in the future?

- **Very certainly not**
- **Certainly not**
- **Maybe yes, maybe not**
- **Certainly yes**
- **Very certainly yes**

Could you explain your answer?

What do you think is needed at organizational level to give interconception care a place in PCHC?

Imagine that in the future interconception care will be integrated in PCHC. To what extent do you find the following types of care suitable?

	Very certainly not	Certainly not	Maybe not, maybe yes	Certainly yes	Very certainly yes
providing information materials					
providing general advice during routine PCHC visits					
screening for risk factors and discussing these during routine visits					
performing a pre-pregnancy/ICC consultation					
discussing referral for ICC at GPs or midwives					
discussing referral for a separate ICC consultation within PCHC					

Interconception care - statement

This is the last page of this questionnaire.

	Strongly disagree	Dis-agree	neutral	agree	Strongly agree
As far as I know, interconception care is based on empirical evidence					
Interconception care is too complicated for me to provide					
ICC is in line with how I am used to work					
I think it is important to contribute to ICC					
I think it is my job to provide ICC					
I have sufficient knowledge and skills to be able to provide ICC					
I find interconception care suitable for my clients					
I expect that clients will generally be satisfied if I provide ICC					
I expect that clients will generally cooperate if I provide ICC					

The end of the questionnaire

Thank you so much for completing the questionnaire.
