# Supplementary material for: Integrating interconception care in preventive child health care services: The Healthy Pregnancy 4 All program
Source: PLoS One. 2019 Nov 6;14(11):e0224427. doi: 10.1371/journal.pone.0224427 (PMC6834275; doi:10.1371/journal.pone.0224427)
Supplement: S4 Questionnaire — (PDF) [file pone.0224427.s006.pdf]

Zorgverleners interconceptiezorg HP4All-2 (vragenlijst 2)

Geachte collega,

Bij voorbaat hartelijk dank voor het invullen van deze vragenlijst.

Hieronder volgt een korte toelichting op de vragenlijst:

Het doel van deze vragenlijst is om te inventariseren hoe JGZ (0-4) zorgverleners aankijken tegen interconceptiezorg, hoe zij interconceptiezorg ervaren binnen de organisatie waar ze werken en hoe zij dit in de toekomst zien.

Onder interconceptiezorg verstaan we alle mogelijke vormen van zorg in voorbereiding op een volgende zwangerschap. U kunt denken aan het meegeven van informatie over gezond zwanger worden en het geven van advies over stoppen met roken of het gebruik van foliumzuur. Ook kunt u verwijzen naar een individueel kinderwensconsult, of deze zelf geven. Op een **kinderwensconsult** wordt persoonlijk advies en begeleiding gegeven ter voorbereiding op een zwangerschap.

Het invullen van de vragenlijst duurt ongeveer 10 minuten.

Lees de vragen zoals ze voor u persoonlijk van toepassing zijn en beantwoord alle vragen. U kunt geen foute antwoorden geven. Het gaat om uw mening.

Basiskarakteristieken

Wat is uw leeftijd? *

Hoeveel jaren werkervaring heeft u in uw huidige functie? *

Interconceptiezorg - bekendheid

In hoeverre vindt u dat u op de hoogte bent van wat interconceptiezorg inhoudt? *

Kies één van de volgende mogelijkheden:

- Ik had voor het lezen van de inleiding geen idee van wat interconceptiezorg inhield
- Ik had er al wel eens van gehoord, maar kan niet benoemen wat het inhoudt
- Ik weet in grote lijnen wat interconceptiezorg inhoudt, maar ken geen inhoudelijke details
- Ik ben redelijk op de hoogte en ken inhoudelijke details
- Ik ben goed op de hoogte van de inhoud en zou dit kunnen uitleggen aan iemand

Huidige situatie

De volgende vragen gaan over in hoeverre er op uw werkplek aandacht wordt besteed aan het verlenen van interconceptiezorg. Denk hierbij aan flyers over gezond zwanger worden, scholingen die worden aangeboden over dit onderwerp, gesprekken met collega's over zorg verlenen aan vrouwen met een kinderwens etcetera. *

Kies het toepasselijke antwoord voor elk onderdeel:

	zeer weinig	weinig	niet weinig, niet veel	veel	zeer veel
**In hoeverre wordt er op uw locatie aandacht besteed aan interconceptiezorg?**					
**In hoeverre denkt u dat uw directe collega's aandacht besteden aan het verlenen van interconceptiezorg?**					
**In hoeverre besteedt u zelf aandacht aan het verlenen van interconceptiezorg?**					

In hoeverre voert u interconceptiezorg taken <u>nu</u> uit? Bij hoeveel clienten? *

Kies het toepasselijke antwoord voor elk onderdeel:

	geen enkele	een minderheid	de helft	een meerderheid	iedereen
**Bespreken van een kinderwens**					
**Materialen meegeven met informatie**					
**Algemene inhoudelijke informatie en adviezen geven**					
**Informeren over de mogelijkheid van een kinderwensconsult**					
**Verwijzen naar een aparte afspraak voor een kinderwensconsult (bij een kinderwens)**					
**Verrichten van een kinderwensconsult (bij een kinderwens)**					

Indien het u niet lukt om interconceptiezorg taken uit te voeren, kunt u dan aangeven waardoor dat voornamelijk komt? *

Selecteer alle mogelijkheden:

- geen tijd vanwege mijn andere taken
- geen tijd omdat de client te laat komt
- ik ervaar onvoldoende expertise
- ik vind het niet mijn taak
- het voelt niet goed vanwege de persoonlijke situatie van de client
- de cliënt staat er niet voor open
- moeizame communicatie met de client (zoals een taalbarriere of lage gezondheidsvaardigheden)
- ik ben het vergeten om uit te voeren
- andere reden

Ervan uitgaande dat het JGZ consult wel heeft plaatsgevonden.

Interconceptiezorg - toekomst

De volgende vragen gaan over uw mening ten opzichte van interconceptiezorg als onderdeel van de JGZ.

In hoeverre acht u het wenselijk dat de JGZ in de toekomst een vorm van interconceptiezorg zal gaan verlenen? *

Kies één van de volgende mogelijkheden:

- Zeer zeker niet
- Zeker niet
- Misschien wel, misschien niet
- Zeker wel
- Zeer zeker wel

Kunt u uw antwoord toelichten?

Vul uw antwoord hier in:

In hoeverre verwacht u dat de JGZ daadwerkelijk in de toekomst interconceptiezorg zal gaan verlenen? *

Kies één van de volgende mogelijkheden:

- Zeer zeker niet
- Zeker niet
- Misschien wel, misschien niet
- Zeker wel
- Zeer zeker wel

Kunt u uw antwoord toelichten?

Vul uw antwoord hier in:

Wat denkt u dat er op organisatieniveau nodig is om interconceptiezorg een plek te geven in de JGZ? *

Vul uw antwoord hier in:

Denk bijvoorbeeld aan afspraken, middelen etc

Stel in de toekomst wordt interconceptiezorg een onderdeel van de JGZ. In hoeverre vindt u dan de onderstaande vormen geschikt? *

Kies het toepasselijke antwoord voor elk onderdeel:

	Zeer zeker niet	Zeker niet	Misschien wel, misschien niet	Zeke r wel	Zeer zeker wel
**Zelf materialen meegeven met informatie of verwijzen naar websites**					
**Zelf algemene inhoudelijke adviezen geven tijdens een standaard consult**					
**Zelf screenen op risicofactoren en deze bespreken tijdens een standaard consult**					
**Zelf een kinderwensconsult verrichten tijdens een standaard consult of op een ander moment**					
**Verwijzen naar verloskundige/huisarts voor een kinderwensconsult***					
**Verwijzen voor een kinderwensconsult binnen uw JGZ-instelling****					

*Verwijzen is in deze context een vrij breed begrip, omdat er niet daadwerkelijk met een verwijzing gewerkt kan worden. **Ervan uitgaande dat dit een bestaande mogelijkheid is in de toekomst.

Interconceptiezorg - stellingen

Dit is de laatste pagina van deze vragenlijst.

Stellingen *

Kies het toepasselijke antwoord voor elk onderdeel:

	helemaal mee oneens	mee oneen s	noch mee oneens, noch mee eens	mee eens	helemaal mee eens
**Interconceptiezorg is voor zover ik weet, gebaseerd op wetenschappelijke kennis**					
**Interconceptiezorg is te ingewikkeld voor mij om te geven**					
**Interconceptiezorg sluit aan bij hoe ik gewend ben om te werken**					

Ik vind het belangrijk om een bijdrage te leveren aan interconceptiezorg

Ik vind het tot mijn functie behoren om interconceptiezorg te geven

Ik beschik over voldoende kennis en vaardigheden om interconceptiezorg te kunnen geven

Ik vind interconceptiezorg geschikt voor mijn clienten

Ik verwacht dat cliënten over het algemeen tevreden zullen zijn als ik interconceptiezorg geef

Ik verwacht dat cliënten over het algemeen zullen meewerken als ik interconceptiezorg geef

Einde

Ontzettend bedankt voor het invullen van de vragenlijst.
