# Supplementary material for: Integrating interconception care in preventive child health care services: The Healthy Pregnancy 4 All program
Source: PLoS One. 2019 Nov 6;14(11):e0224427. doi: 10.1371/journal.pone.0224427 (PMC6834275; doi:10.1371/journal.pone.0224427)
Supplement: S5 Questionnaire — (PDF) [file pone.0224427.s007.pdf]

Questionnaire 1

Study into the pre-pregnancy clinics and the preparation for a subsequent pregnancy.

This questionnaire forms part of the **Healthy Pregnancy 4 All Project**.

We are pleased that you want to complete the questionnaire. The results will contribute to improving the care prior to a pregnancy.

The questionnaire consists of 3 sections. The questions are about:

- General data (such as your background, lifestyle and health)

- Your previous pregnancy/pregnancies and your desire to have a child

- The preparation for a subsequent pregnancy and the pre-pregnancy clinic.

There is space for you to write comments at the end of the questionnaire.

It will take you about 20 minutes to fill in the questionnaire.

Please answer all the questions.

If you are in doubt, choose the answer that is closest to your situation.

Your answers will be treated confidentially and processed anonymously using a code. We do not ask you for your name. We do, however, ask for the numbers in your postal code.

Your healthcare provider (for example, the doctor or nurse at the Child Health Clinic, your midwife or GP) will not see the answers you write on this questionnaire. Therefore, your healthcare provider may ask you the same questions once more if you go to the pre-pregnancy clinic.

The HP4All-2 team

General - registration

1.What are the four numbers of your postal code? ________________________

2.What is your (youngest) child's date of birth? ________________________

3.What is your date of birth? ________________________

4.Can you indicate in which country you, your mother and your father were born?

	The Netherlands	Suriname	The Netherlands Antilles/Aruba/Curaçao	Morocco	Turkey	Middle or Eastern Europe	Cape Verde	Indonesia/The Moluccas	Other
You	○	○	○	○	○	○	○	○	○
Your mother	○	○	○	○	○	○	○	○	○
Your father	○	○	○	○	○	○	○	○	○

5. Which population group do you consider yourself part of?

Dutch	Suriname-Creole	Suriname-Hindustani	Suriname-other	Antillean/Aruban/Curaçao	Moroccan	Turkish	Middle or Eastern European	Cape Verdian	Indonesian/Moluccan	Other
○	○	○	○	○	○	○	○	○	○	○

General - education and work

6. Are you able to read Dutch?

Select one of the following options:

- Yes, without problems
- Yes, a little bit
- No

7.If you visit a healthcare provider, are you able to understand what he/she tells you?

- Always
- Often
- Sometimes
- Rarely

By healthcare provider, we mean for example a GP, midwife or nurse

8.What is the highest level of education that you have completed?

Select one of the following options:

- Primary school
- Special education, learning difficulties (children with learning difficulties [MLK], children with severe learning difficulties [ZMLK], severely maladjusted children [ZMOK])
- Preparatory vocational education (VBO)/preparatory vocational secondary education (VMBO) basic vocational or advanced vocational track
- Junior general secondary education (MAVO)/preparatory vocational secondary education (VMBO) combined or theoretical track
- Senior general secondary education (HAVO)
- Pre-university education (VWO)
- Senior secondary vocational education (MBO)
- Higher professional education (HBO)
- University education (university/post HBO)
- Other

9. Do you have a paid job at the moment?

- Yes
- No

10.What is your <u>family's</u> net income per month?

- Less than 1,000 euro per month
- 1,000 up to and including 1,499 euro per month
- 1,500 up to and including 1,999 euro per month
- 2,000 up to and including 2,499 euro per month
- 2,500 up to and including 3,000 euro per month
- More than 3,000 euro per month

'Net income' is the amount transferred to your account or which you actually receive. Add up the income of all the people in your household, including your partner.

11. Do you have financial problems or debts which are difficult to pay off at the moment?

- Yes
- No

12. Do you have healthcare insurance at the moment?

- Yes, only the basic insurance package
- Yes, a basic and an additional insurance package
- No, I do not have healthcare insurance

<u>General - social environment</u>

13. Do you have a partner at the moment?

Select one of the following options:

- Yes, and we live together
- Yes, but we do NOT live together
- No, I do not have a partner

14. How many living children do you have? ________________________________

15. Do you make use of childcare?

- Yes, by family or friends
- Yes, by a paid organisation
- Yes, by a combination of a paid organisation and family or friends
- No
- Other

16. Do you have relationship problems, for example with your partner or family, at the moment?

- Yes, with my partner
- Yes, with my family
- Yes, with friends
- No
- Other

17. Do you feel that you are getting enough support from those around you? By this we mean from your partner, family and friends.

- Yes
- No

General - lifestyle and medical

18. Do you smoke?

- Yes, every day
- Yes, but only now and again
- No, I have never smoked
- No, but I did in the past

19. How much do you smoke on a normal day? ______________________________

For example, how many *cigarettes* do you smoke per day?

20. Does your partner smoke?

- Yes
- No

21. Do you drink alcohol?

- Yes, every day
- Yes, but only now and again
- No, I have never drunk alcohol
- No, but I did in the past

22. Do you use drugs?

- Yes, every day
- Yes, but only now and again
- No, I have never used drugs
- No, but I did in the past

23. What applies to you in relation to your previous pregnancy?

- I smoked during my previous pregnancy
- I drank alcohol during my previous pregnancy
- I used drugs during my previous pregnancy
- None of the above apply

24. Are you taking folic acid tablets at the moment?

- Yes, every day
- Yes, but only now and again
- No

25. What applies to you in relation to your previous pregnancy?

- I took folic acid prior to my previous pregnancy
- I took folic acid during my previous pregnancy
- I took folic acid prior to and during my previous pregnancy
- I did NOT take folic acid during my previous pregnancy

26. Do you take any medicines that you can buy without a prescription (for example at a chemist) at the moment?

- Yes
- I do not know whether I take any non-prescription medicines
- No

27. Which medicines that you can buy without a prescription do you use?

- Vitamin pills (suitable for pregnancy and if you want to become pregnant)
- Painkillers
- Medicines for colds
- Tranquillisers or sleeping pills
- Homeopathic medicines
- Other

28. Are you using any prescription medicines (prescribed by a doctor) at the moment?

- Yes
- I do not know whether I am taking any medicines
- No

29. Which prescription medicines are you using?

- Iron tablets
- Antibiotics
- Anti-inflammatories/painkillers
- Medicines to treat high blood pressure
- Medicines to treat nausea
- Medicines for the thyroid gland
- Tranquillisers or sleeping pills
- Medicines for depression
- Insulin
- Other

30. Do you have a chronic illness for which you go to a doctor or take medicines?

- Yes
- I do not know whether I have a chronic illness
- No

31. What chronic illness do you have?

- High blood pressure (hypertension)
- Diabetes
- Thyroid disorder
- Kidney and/or cardiovascular (heart and blood vessels) disease
- Epilepsy
- Psychiatric disorder
- Other

<u>Your pregnancies and desire to become pregnant</u>

The following questions apply to your situation at the moment (including your previous pregnancy).

32. How many times have you been pregnant?

.....times

33. Did you have any problems in your previous pregnancy/pregnancies? Such as diabetes, high blood pressure or pre-eclampsia?

Select one of the following options:

- Yes, I had diabetes, high blood pressure or pre-eclampsia
- No
- Other

34. Have you ever had a miscarriage?

- Yes, once
- Yes, twice
- Yes, more than 2
- No

35. Have you ever had an abortion?

- Yes
- No

36. Was (one of) your child(ren) born via a Caesarean section or suction cup (vacuum pump) or forceps delivery?

- Yes, by Caesarean section
- Yes, using a suction cup or forceps
- Yes, I have had both a delivery using suction cup or forceps and a Caesarean section
- No

37. Did you experience one of the following problems in your previous pregnancy/pregnancies?

	Yes	No
Was (one of) your child(ren) born too early? Before about 8.5 months (37 weeks) of pregnancy?	◯	◯
Was (one of) your child(ren) born weighing less than 2,500 grams?	◯	◯
Was (one of) your child(ren) born weighing more than 4,500 grams?	◯	◯
Does (one of) your child(ren) have a birth defect?	◯	◯
Did (one of) your child(ren) have a poor start following the birth (for example, blue or limp) for which the paediatrician had to assist or the baby needed help with breathing?	◯	◯
Have you ever had a child that died in the period surrounding the birth?	◯	◯

38. Which of the following applies to you? Select the sentence below that best fits the *feeling* that you have about your previous pregnancy/pregnancies and/or delivery/deliveries.

- Not a good feeling at all
- A slightly positive feeling
- A reasonably positive feeling
- A good feeling
- A very good feeling

39. When do you hope or expect to become pregnant again?

- I am pregnant at the moment
- Between now and 3 months
- Within 3 to 6 months
- Within 6 months to a year
- Within one to 2 years
- In 2 years or more
- I do not know yet when I want to become pregnant again
- I have been advised not to become pregnant again (for the time being)
- I do not want to become pregnant again

40. Does the feeling that you have about your previous pregnancy/pregnancies and/or delivery/deliveries play a role in the *moment* at which you would like to become pregnant again?

- o Yes, it means that I am not sure yet whether or not I want to become pregnant again
- o Yes, it means that I am delaying another pregnancy for a while
- o Yes, it means that I do not want to wait very long with becoming pregnant again
- o No, it does not play a role

41. Do you or your partner use contraceptives?

- o Yes
- o No

Preparation for a subsequent pregnancy and the Pre-Pregnancy Clinic

Knowledge

There are issues that can increase the chances of a healthy start to the pregnancy and the chances of a healthy baby. There are also issues that can form a risk to the pregnancy and the baby.

42. What do you think is true and false?

	True	False	I do not know
If you smoke, it will take longer to become pregnant	○	○	○
If you smoke during the pregnancy, the risk of premature birth is higher	○	○	○
Folic acid is good for the baby's growth and development	○	○	○
The best time to start taking folic acid tablets is as soon as you have become pregnant	○	○	○

Intentions pre-pregnancy clinic and advice

You have heard at the Child Health Clinic that you can be examined and receive advice from a healthcare provider even before you become pregnant. We now refer to this as the PRE-PREGNANCY CLINIC.

43. We would like to know your opinion on the pre-pregnancy clinic.

Even if you do not want to become pregnant (yet), we would still like you to answer all the questions!

	I agree entirely	I agree	Neutral	I disagree	I disagree entirely
If I want to become pregnant again, then I would definitely visit a free pre-pregnancy clinic beforehand	◯	◯	◯	◯	◯
If I want to become pregnant again, then I would definitely visit a pre-pregnancy clinic beforehand if it costs € 15	◯	◯	◯	◯	◯
If I want to become pregnant again, then I would definitely stop smoking before becoming pregnant again	◯	◯	◯	◯	◯
If I want to become pregnant again, then I would definitely take a folic acid tablet every day before becoming pregnant again	◯	◯	◯	◯	◯

Further to the left: then you "**agree** entirely". Further to the right: then you "**disagree** entirely".

Attitude towards the pre-pregnancy clinic

The aim of a pre-pregnancy clinic is to have the healthiest possible start to the pregnancy.

44. We would like to know what you think about it.

	I agree entirely	I agree	Neutral	I disagree	I disagree entirely
A Pre-Pregnancy Clinic before the start of a pregnancy is not necessary	◯	◯	◯	◯	◯
A Pre-Pregnancy Clinic must be advertised everywhere on posters. For example, at the GP, the midwife, the Child Health Clinic, at tram/bus stops, in shops and in public buildings	◯	◯	◯	◯	◯
A Pre-Pregnancy Clinic should be accessible free of charge for everyone who wants to become pregnant	◯	◯	◯	◯	◯
If you visit a Pre-Pregnancy Clinic, then you will know how to achieve a healthy pregnancy	◯	◯	◯	◯	◯

Who do you receive advice from?

45. Who is important to you when deciding whether to visit a pre-pregnancy clinic prior to the pregnancy?

	I agree entirely	I agree	Neutral	I disagree	I disagree entirely
My partner's opinion is important to me when deciding to go to a pre-pregnancy clinic	○	○	○	○	○
My family's opinion is important to me when deciding to go to a pre-pregnancy clinic	○	○	○	○	○
The opinion of my friends/acquaintances is important to me when deciding to go to a pre-pregnancy clinic	○	○	○	○	○
The opinion of my healthcare providers (such as GP, midwife, gynaecologist, child health physician or nurse) is important to me when deciding to go to a pre-pregnancy clinic	○	○	○	○	○

Own Effort

46. You may already receive advice from a midwife, doctor or nurse before the pregnancy. We would like to know how difficult you find it to follow this advice.

	Very difficult	Fairly difficult	Fairly easy	Very easy
If you (were to) smoke: what would it be like for you to stop smoking?	◯	◯	◯	◯
How easy/difficult is it for you to take a pill (folic acid) every day?	◯	◯	◯	◯
How easy/difficult is it for you to visit a pre-pregnancy clinic?	◯	◯	◯	◯
How easy/difficult is it for you to discuss your desire for a child with a healthcare provider?	◯	◯	◯	◯

Preparation for a pregnancy and the Pre-Pregnancy Clinic.

Barriers to attending the pre-pregnancy clinic

47. We would like to know why you might prefer not to go to the pre-pregnancy clinic

	I agree entirely	I agree	Neutral	I disagree	I disagree entirely
It takes too much time and effort to visit a pre-pregnancy clinic	◯	◯	◯	◯	◯
I dread going to the pre-pregnancy clinic	◯	◯	◯	◯	◯
I will not benefit enough from going to a pre-pregnancy clinic	◯	◯	◯	◯	◯
I am worried about negative reactions from my husband or family if I go to a pre-pregnancy clinic	◯	◯	◯	◯	◯
My religion or personal beliefs say that I should not go to a pre-pregnancy clinic	◯	◯	◯	◯	◯

48. What is the most important reason for you not to visit a Pre-Pregnancy Clinic?

49. What is the most important reason for deciding that you would visit a Pre-Pregnancy Clinic?

- I would like to receive information/I want to prepare for a subsequent pregnancy
- Following advice from the Child Health Clinic
- Following advice from the midwife, gynaecologist or GP
- My partner wants me to go
- Following advice from my family/friends
- The outcome of a previous pregnancy was not what I wanted
- I have a child with a condition
- I see no reason to go
- Other:

Statements about the baby's health and illness

50. Below we want to find out what you think you can do yourself to have a healthy baby

	I agree entirely	I agree	Neutral	I disagree	I disagree entirely
There is nothing that I can do to ensure that my baby is born healthy	○	○	○	○	○
It is my job as a mother to ensure that my baby is born healthy	○	○	○	○	○
There are few choices that I can make that will affect the health of my baby at his/her birth	○	○	○	○	○
There is a lot that I can do to ensure that my baby is born healthy	○	○	○	○	○
There are things that I can do before I become pregnant to ensure that my baby is born healthy	○	○	○	○	○

Preparation for a pregnancy and the Pre-Pregnancy Clinic - experiences and expectations

Your experiences during the conversation about your desire to have a child and the pre-pregnancy clinic

Your desire to have a child and the pre-pregnancy clinic were discussed at the Child Health Centre.

51. We would like to know how you experienced this.

	I agree entirely	I agree	Neutral	I disagree	I disagree entirely
I received personal attention during this conversation	◯	◯	◯	◯	◯
I was given the option to decide for myself whether or not to go to the pre-pregnancy clinic	◯	◯	◯	◯	◯
My privacy was respected	◯	◯	◯	◯	◯
It was explained to me why my desire to have a child and the pre-pregnancy clinic were being discussed	◯	◯	◯	◯	◯
I think that it is good that I was asked whether I want to have a(nother) child	◯	◯	◯	◯	◯

52. How soon do you think that you should be able to visit a pre-pregnancy clinic?

- As soon as possible, within 2 weeks
- Within 2 months
- Within 6 months
- Within one year
- Only after a year

53. Do you think that your partner should be included in the pre-pregnancy clinic?

- Yes
- No
- I do not have a partner

54. How do you think that you should receive information about the Pre-Pregnancy Clinic?

	Yes	Maybe	No
Internet	◯	◯	◯
Library	◯	◯	◯
Community Centre	◯	◯	◯

Posters	◯	◯	◯
Information leaflet	◯	◯	◯
Via the midwife/gynaecologist	◯	◯	◯
Via the GP	◯	◯	◯
Via the Child Health Clinic	◯	◯	◯
Via school	◯	◯	◯
Via work	◯	◯	◯
Via friends/family	◯	◯	◯

55. Is there any other way in which you would like to receive information about the pre-pregnancy clinic?

- No
- Other

56. Which healthcare provider would you like to visit for advice about a subsequent pregnancy?

	Yes	Maybe	No
GP	◯	◯	◯
Midwife	◯	◯	◯
Gynaecologist	◯	◯	◯
Paediatrician	◯	◯	◯
Doctor at the Child Health Clinic	◯	◯	◯
Nurse at the Child Health Clinic	◯	◯	◯

57. Is there anyone else who you would like to visit for advice about a subsequent pregnancy?

- No
- Other:

58. Have you visited a pre-pregnancy clinic before?

- Yes
- No

This is the end of the questionnaire.
Thank you for filling in this questionnaire.
You will receive questionnaire 2, a short questionnaire, about half a year from now

Space for general comments:
