# Supplementary material for: Integrating interconception care in preventive child health care services: The Healthy Pregnancy 4 All program
Source: PLoS One. 2019 Nov 6;14(11):e0224427. doi: 10.1371/journal.pone.0224427 (PMC6834275; doi:10.1371/journal.pone.0224427)
Supplement: S6 Questionnaire — (PDF) [file pone.0224427.s008.pdf]

Deze vragenlijst hoort bij het project Healthy Pregnancy 4 All-2
(Een Gezonde Zwangerschap voor Iedereen).

Wij zijn u dankbaar dat u de vragenlijst wilt invullen. De resultaten zullen bijdragen aan het verbeteren van de zorg voorafgaand aan een zwangerschap.

De vragenlijst bestaat uit 3 onderdelen. De vragen gaan over:

1. Algemene gegevens (zoals uw achtergrond, leefstijl en gezondheid)
2. Uw vorige zwangerschap(pen) en uw kinderwens
3. De voorbereiding op een eventuele volgende zwangerschap en het kinderwensspreekuur.

Aan het einde van de vragenlijst is er ruimte om opmerkingen te plaatsen.

Het invullen van de vragenlijst duurt ongeveer 20 minuten.

Wij verzoeken u om alle vragen te beantwoorden.

Als u twijfelt, kies dan het antwoord dat het beste bij u past.

De gegevens uit deze vragenlijsten worden vertrouwelijk behandeld en anoniem met een code verwerkt. Wij vragen niet naar uw naam. Wel vragen wij om uw postcode cijfers.

Uw zorgverlener (zoals bijvoorbeeld uw consultatiebureau arts of verpleegkundige, uw verloskundige of huisarts) ziet de antwoorden die u heeft ingevuld in deze vragenlijst niet. Het kan dus voorkomen, dat uw zorgverlener nog een keer dezelfde vragen aan u stelt als u naar een kinderwensspreekuur gaat.

Het HP4All-2 team

**Studienummer**	☐☐ – ☐☐ – ☐☐☐☐
**Geboortedatum**	☐☐ – ☐☐ – ☐☐☐☐

Sectie A: Algemeen - registratie

A1. Wat zijn de vier cijfers van uw postcode?

☐☐☐☐

A2. Wat is de geboortedatum van uw (jongste) kind?

☐☐☐☐☐☐☐☐

A3. Wat is uw geboortedatum?

☐☐☐☐☐☐☐☐

A4. Kunt u aangeven in welk land u, uw moeder en uw vader geboren zijn?

	Nederland	Suriname	Nederlandse Antillen/ Aruba/ Curaçao	Marokko	Turkije	Midden- of Oost-Europa	Kaapverdië	Indonesië/ Molukken	Anders
Uzelf	☐	☐	☐	☐	☐	☐	☐	☐	☐
Uw moeder	☐	☐	☐	☐	☐	☐	☐	☐	☐
Uw vader	☐	☐	☐	☐	☐	☐	☐	☐	☐

A5. Tot welke bevolkingsgroep rekent u zichzelf?

Neder-lands	Suri-naams-Creools	Surinaams-Hindoestaans	Surinaams-overig	Antilliaans/ Arubaans/ Curaçaos	Marok-kaans	Turks	Midden- of Oost-Europees	Kaap-verdisch	Indonesisch/ Moluks	Anders
☐	☐	☐	☐	☐	☐	☐	☐	☐	☐	☐

Sectie B: Algemeen - opleiding en werk

B1. Kunt u Nederlands lezen?

- Ja, zonder problemen ☐
- Ja, een klein beetje ☐
- Nee ☐

B2. Als u bij een zorgverlener komt, begrijpt u dan wat zij of hij vertelt?

Met zorgverlener bedoelen we bijvoorbeeld een arts, verloskundige of verpleegkundige

- Altijd ☐
- Vaak ☐
- Soms ☐
- Zelden ☐

B3. Wat is de hoogste opleiding die u hebt afgemaakt?

- Lagere school / basisonderwijs ☐
- Speciaal onderwijs, leerproblemen (MLK, ZMLK, ZMOK) ☐
- VBO / VMBO beroeps- of kadergerichte leerweg ☐
- MAVO / VMBO gemengd of theoretische leerweg ☐
- HAVO ☐
- VWO ☐
- Middelbaar beroepsonderwijs (MBO) ☐
- Hoger beroepsonderwijs (HBO) ☐
- Wetenschappelijk onderwijs (universiteit, post-hbo) ☐
- Andere ☐

Andere

B4. Heeft u op dit moment een betaalde baan?

- Ja ☐
- Nee ☐

B5. Wat is het netto inkomen van uw gezin per maand?

Netto inkomen is het bedrag dat u op uw rekening of in handen krijgt. Reken het inkomen van alle mensen in uw huishouden mee, dus ook van uw partner met een inkomen.

- Minder dan 1000 euro per maand ☐
- 1000 tot en met 1499 euro per maand ☐
- 1500 tot en met 1999 euro per maand ☐
- 2000 tot en met 2499 euro per maand ☐
- 2500 tot en met 3000 euro per maand ☐
- Meer dan 3000 euro per maand ☐

B6. Heeft u op dit moment financiële problemen of moeilijk aflosbare schulden?

- Ja ☐
- Nee ☐

B7. Heeft u op dit moment een ziektenkosten verzekering?

- Ja, alleen een basisverzekering ☐
- Ja, een basis en aanvullende verzekering ☐
- Nee, geen ziektekostenverzekering ☐

Sectie C: Algemeen - sociale omgeving

C1. Heeft u op dit moment een partner?

- Ja, een partner met wie ik samenwoon ☐
- Ja, een partner met wie ik NIET samenwoon ☐
- Nee, ik heb geen partner ☐

C2. Hoeveel levende kinderen heeft u?

☐☐

C3. Maakt u gebruik van kinderopvang?

- Ja, door familie of vrienden ☐
- Ja, door een betaalde organisatie ☐
- Ja, door een combinatie van een betaalde organisatie en familie of vrienden ☐
- Nee ☐
- Andere ☐

Andere

C4. Heeft u op dit moment relationele problemen met bijvoorbeeld uw partner of familie?

- Ja, met mijn partner ☐
- Ja, met familie ☐
- Ja, met vrienden ☐
- Nee ☐
- Andere ☐

Andere

C5. Ervaart u voldoende steun uit uw omgeving? Hiermee bedoelen we van uw partner, familie en vrienden.

- Ja ☐
- Nee ☐

Sectie D: Algemeen - leefstijl en medisch

D1. Rookt u?

- Ja, dagelijks ☐
- Ja, af en toe ☐
- Nee, ik heb nooit gerookt ☐
- Nee, maar wel in het verleden ☐

D2. Hoeveel rookt u op een normale dag?

Bijvoorbeeld hoeveel sigaretten op een dag

☐☐

D3. Rookt uw partner?

- Ja ☐
- Nee ☐

D4. Drinkt u alcohol?

- Ja, dagelijks ☐
- Ja, af en toe ☐
- Nee, ik heb nooit alcohol gedronken ☐
- Nee, maar wel in het verleden ☐

D5. Gebruikt u drugs?

- Ja, dagelijks ☐
- Ja, af en toe ☐
- Nee, ik heb nooit drugs gebruikt ☐
- Nee, maar wel in het verleden ☐

D6. Wat is voor u van toepassing als het gaat over uw vorige zwangerschap?

- Ik heb gerookt in mijn vorige zwangerschap ☐
- Ik heb alcohol gedronken in mijn vorige zwangerschap ☐
- Ik heb drugs gebruikt in mijn vorige zwangerschap ☐
- Geen van bovenstaande van toepassing ☐

D7. Gebruikt u nu foliumzuur tabletten?

- Ja, dagelijks ☐
- Ja, af en toe ☐
- Nee ☐

D8. Wat is voor u van toepassing als het gaat over uw vorige zwangerschap

- Ik heb foliumzuur gebruikt voorafgaand aan mijn vorige zwangerschap ☐
- Ik heb foliumzuur gebruikt tijdens mijn vorige zwangerschap ☐
- Ik heb foliumzuur gebruikt voorafgaand en tijdens mijn vorige zwangerschap ☐
- Ik heb geen foliumzuur gebruikt in mijn vorige zwangerschap ☐

D9. Gebruikt u op dit moment medicijnen die u zonder recept kunt kopen (bijvoorbeeld bij de drogist)?

- Ja ☐
- Ik weet niet of ik medicijnen zonder recept gebruik ☐
- Nee ☐

D10. Welke medicijnen gebruikt u die u zonder recept kunt kopen?

- Vitaminepillen (geschikt bij kinderwens en zwangerschap) ☐
- Pijnstillers ☐
- Middelen tegen verkoudheid ☐
- Pillen voor kalmering en slapen ☐
- Homeopatische middelen ☐
- Andere ☐

Andere

D11. Gebruikt u op dit moment medicijnen op recept/voorschrift van een arts?

- Ja ☐
- Ik weet niet of ik medicijnen gebruik ☐
- Nee ☐

D12. Welke medicijnen gebruikt u op recept?

- Staal-/ijzerpillen ☐
- Antibiotica ☐
- Ontstekingsremmers/pijnstillers ☐
- Medicijnen tegen hoge bloeddruk ☐
- Medicijnen tegen misselijkheid ☐
- Medicijnen voor mijn schildklier ☐
- Medicijnen voor kalmering en slapen ☐
- Medicijnen tegen depressie ☐
- Insuline ☐
- Andere ☐

Andere

D13. Heeft u een chronische ziekte waarvoor u naar een arts gaat of medicijnen gebruikt?

- Ja ☐
- Ik weet niet of ik een chronische ziekte heb ☐
- Nee ☐

D14. Welke chronische ziekte?

- Hoge bloeddruk (hypertensie) ☐
- Suikerziekte (diabetes) ☐
- Schildklier aandoening ☐
- Nierziekte en/of hart- en vaatziekte ☐
- Epilepsie ☐
- Psychische aandoening ☐
- Andere ☐

Andere

Sectie E: Uw zwangerschappen en kinderwens

De volgende vragen gaan over uw situatie op dit moment (uw afgelopen zwangerschap inbegrepen).

E1. Hoe vaak bent u zwanger geweest?

E2. Heeft u in uw vorige zwangerschap(pen) problemen gehad? Zoals suikerziekte, hoge bloeddruk of zwangerschapsvergiftiging?

- Ja, ik heb suikerziekte, hoge bloeddruk of zwangerschapsvergiftiging gehad ☐
- Nee ☐
- Andere ☐

Andere

E3. Heeft u ooit een miskraam gehad?

- Ja, 1 keer ☐
- Ja, 2 keer ☐
- Ja, meer dan 2 keer ☐
- Nee ☐

E4. Heeft u ooit een abortus gehad?

- Ja ☐
- Nee ☐

E5. Is (één van) uw kind(eren) met een keizersnede of zuignap (vacuumpomp) of tang geboren?

- Ja, met een keizersnede ☐
- Ja, met een zuignap of tang ☐
- Ja, ik heb zowel een bevalling met een zuignap of tang gehad, als een keizersnede ☐
- Nee ☐

E6. Was er sprake van één van de volgende problemen in uw vorige zwangerschap(pen)?

	Ja	Nee
Is (één van) uw kind(eren) te vroeg geboren? Vóór ongeveer 8,5 maand (37 weken) zwangerschap?	☐	☐
Was (één van) uw kind(eren) lichter dan 2500gram bij de geboorte?	☐	☐
Was (één van) uw kind(eren) zwaarder dan 4500gram bij de geboorte?	☐	☐
Heeft (één van) uw kind(eren) een aangeboren afwijking?	☐	☐
Had (één van) uw kind(eren) een slechte start bij de geboorte (bijvoorbeeld blauw of slap) waarvoor de kinderarts moest komen of beamend moest worden?	☐	☐
Heeft u ooit een kind gekregen dat rond de geboorte is overleden?	☐	☐

E7. Wat geldt voor u? Kies uit onderstaande zinnen wat het beste past bij het *gevoel* dat u heeft overgehouden aan uw zwangerschap(pen) en/of bevalling(en).

- Absoluut geen goed gevoel ☐
- Een enigszins goed gevoel ☐
- Een redelijk goed gevoel ☐
- Een goed gevoel ☐
- Een zeer goed gevoel ☐

E8. Wanneer hoopt of verwacht u opnieuw zwanger te worden?

- Ik ben nu zwanger ☐
- Binnen nu en 3 maanden ☐
- Binnen 3 tot 6 maanden ☐
- Binnen 6 maanden tot een jaar ☐
- Binnen een jaar tot 2 jaar ☐
- Over 2 jaar of langer ☐
- Ik weet nog niet of ik opnieuw zwanger wil worden ☐
- Ik heb het advies gekregen (voorlopig) niet opnieuw zwanger te worden ☐
- Ik wil niet weer zwanger worden ☐

E9. Speelt het gevoel dat u heeft overgehouden aan de vorige zwangerschap(pen) en/of bevalling(en) een rol bij het *moment* waarop u opnieuw zwanger wil worden?

- Ja, het maakt dat ik nog niet weet of ik nog een keer zwanger wil worden ☐
- Ja, het maakt dat ik een volgende zwangerschap nog een tijd uitstel ☐
- Ja, het maakt dat ik niet te lang wil wachten om weer zwanger te worden ☐
- Nee, het speelt geen rol ☐

E10. Gebruikt u zelf of uw partner nu voorbehoedsmiddelen?

- Ja ☐
- Nee ☐

Sectie F: Voorbereiding op een zwangerschap en het Kinderwensspreekuur

F1.

Kennis

Er zijn zaken die de kans op een gezonde start van de zwangerschap en de kans op een gezonde baby vergroten. Ook zijn er zaken die een risico vormen voor de zwangerschap en de baby.

Wat is volgens u waar en wat is niet waar?

	Waar	Niet waar	Weet ik niet
Als je rookt, word je minder snel zwanger	☐	☐	☐
Als je rookt in de zwangerschap, is de kans op vroeggeboorte groter	☐	☐	☐
Foliumzuur is goed voor de groei en ontwikkeling van de baby	☐	☐	☐
Het beste moment om een pilletje foliumzuur te gaan slikken is direct nadat je zwanger bent geworden	☐	☐	☐

F2.

Voornemens kinderwensspreekuur en adviezen

Op het consultatiebureau heeft u gehoord dat u zich al voordat u zwanger bent kunt laten onderzoeken of adviseren door een zorgverlener. Wij noemen dat het kinderwensspreekuur.

Graag willen wij weten wat uw mening over het kinderwensspreekuur is.

Ook als u nu (nog) geen wens tot zwangerschap heeft, willen wij graag uw antwoord weten op alle vragen!

Verder naar links: dan bent u het er 'helemaal mee eens'. Verder naar rechts dan bent u het er 'helemaal mee oneens'.

	Helemaal mee eens	Mee eens	Neutraal	Mee oneens	Helemaal mee oneens
Als ik opnieuw zwanger wil worden, dan zou ik zeker voorafgaand naar een gratis kinderwensspreekuur gaan	☐	☐	☐	☐	☐
Als ik opnieuw zwanger wil worden, dan zou ik zeker voorafgaand naar een kinderwensspreekuur gaan als het €15,- kost	☐	☐	☐	☐	☐

	Helemaal mee eens	Mee eens	Neutraal	Mee oneens	Helemaal mee oneens
Als ik opnieuw zwanger wil worden, dan zou ik zeker stoppen met roken voordat ik weer zwanger word	☐	☐	☐	☐	☐
Als ik opnieuw zwanger wil worden, dan zou ik zeker elke dag een pilletje foliumzuur slikken voordat ik weer zwanger word	☐	☐	☐	☐	☐

F3.

Houding tegenover het kinderwensspreekuur

Het doel van een kinderwensspreekuur is om zo gezond mogelijk aan de zwangerschap te beginnen.

Wij willen graag weten wat u daarvan vindt.

	Helemaal mee eens	Mee eens	Neutraal	Mee oneens	Helemaal mee oneens
Een kinderwensspreekuur voor aanvang van een zwangerschap is niet nodig	☐	☐	☐	☐	☐
Een kinderwensspreekuur moet overal op posters te zien zijn. Bijvoorbeeld bij de huisarts, de verloskundige, het consultatiebureau, bij tramhaltes, in winkels en in openbare gebouwen	☐	☐	☐	☐	☐
Een kinderwensspreekuur moet voor iedereen die zwanger wil worden gratis beschikbaar zijn	☐	☐	☐	☐	☐
Als je een kinderwensspreekuur bezoekt dan weet je daarna hoe je gezond zwanger kunt worden	☐	☐	☐	☐	☐

F4. Door wie laat u zich adviseren

Wie is voor u belangrijk bij de beslissing om voorafgaand aan de zwangerschap naar een kinderwensspreekuur te gaan?

	Helemaal mee eens	Mee eens	Neutraal	Mee oneens	Helemaal mee oneens
De mening van mijn partner is voor mij belangrijk om naar een kinderwensspreekuur te gaan	☐	☐	☐	☐	☐
De mening van mijn familie is voor mij belangrijk om naar een kinderwensspreekuur te gaan	☐	☐	☐	☐	☐
De mening van mijn vrienden/kennissen is voor mij belangrijk om naar een kinderwensspreekuur te gaan	☐	☐	☐	☐	☐
De mening van mijn zorgverleners (zoals huisarts, verloskundige, gynaecoloog, jeugdarts of verpleegkundige) is voor mij belangrijk om naar een kinderwensspreekuur te gaan	☐	☐	☐	☐	☐

F5.

<u>Eigen Inzet</u>

Het kan zijn dat u al <u>voor</u> de zwangerschap van een verloskundige, arts of verpleegkundige adviezen krijgt. Graag willen wij weten hoe moeilijk u het vindt om die op te volgen.

	Heel moeilijk	Beetje moeilijk	Beetje makkelijk	Heel makkelijk
Als u (zou) rookt (roken): hoe is het voor u om te stoppen met roken?	☐	☐	☐	☐
Hoe is het voor u om elke dag een pilletje (foliumzuur) te slikken?	☐	☐	☐	☐
Hoe is het voor u om naar een kinderwensspreekuur te gaan?	☐	☐	☐	☐
Hoe is het voor u om u kinderwens te bespreken met een zorgverlener?	☐	☐	☐	☐

Sectie G: Voorbereiding op een zwangerschap en het Kinderwensspreekuur.

U bent op de een-na-laatste pagina.

G1.

<u>Belemmeringen om naar het kinderwensspreekuur te gaan</u>

Graag willen wij weten waarom u misschien liever <u>niet</u> naar het kinderwensspreekuur gaat

	Helemaal mee eens	Mee eens	Neutraal	Mee oneens	Helemaal mee oneens
Het kost me te veel tijd en moeite om naar een kinderwensspreekuur te gaan	☐	☐	☐	☐	☐
Ik zie op tegen het kinderwensspreekuur	☐	☐	☐	☐	☐
Een kinderwensspreekuur levert mij te weinig op	☐	☐	☐	☐	☐
Ik ben bang voor negatieve reacties van mijn man of familie als ik naar een kinderwensspreekuur ga	☐	☐	☐	☐	☐
Van mijn geloof of levensovertuiging mag ik niet naar een kinderwensspreekuur gaan	☐	☐	☐	☐	☐

G2. Wat is voor u de belangrijkste reden om <u>niet</u> naar een kinderwensspreekuur te gaan?

G3. Wat is voor u de belangrijkste reden om <u>wel</u> naar het kinderwensspreekuur te gaan?

- Ik wil graag informatie/ ik wil me goed voorbereiden op een volgende zwangerschap ☐
- Op advies van het consultatiebureau ☐
- Op advies van de verloskundige, gynaecoloog of huisarts ☐
- Mijn partner wil dit graag ☐
- Op advies van mijn familie / vrienden ☐
- Ik heb een zwangerschap meegemaakt die anders is verlopen dat ik wilde ☐
- Ik heb een kind met een aandoening ☐
- Ik zie geen reden om te gaan ☐
- Andere ☐

Andere

G4. Uitspraken over gezondheid en ziekte van de baby

Hieronder willen wij weten hoe u denkt over wat je zelf kan doen om een gezonde baby te krijgen

	Helemaal mee eens	Mee eens	Neutraal	Mee oneens	Helemaal mee oneens
Er is niets wat ik kan doen om ervoor te zorgen dat mijn baby gezond geboren wordt	☐	☐	☐	☐	☐
Het is mijn taak als moeder om ervoor te zorgen dat mijn baby gezond geboren wordt	☐	☐	☐	☐	☐
Er zijn weinig keuzes die ik kan maken die van invloed zijn op de gezondheid van mijn baby bij de geboorte	☐	☐	☐	☐	☐
Ik kan veel doen om ervoor te zorgen dat mijn baby gezond geboren wordt	☐	☐	☐	☐	☐
Er zijn dingen die kan doen voordat ik zwanger word om ervoor te zorgen dat mijn baby gezond geboren wordt	☐	☐	☐	☐	☐

Sectie H: Voorbereiding op een zwangerschap en het Kinderwensspreekuur - ervaringen en verwachtingen

U bent op de laatste pagina.

H1.

Uw ervaring tijdens het gesprek over uw kinderwens en het kinderwensspreekuur

Op het consultatiebureau is gesproken over uw kinderwens en het kinderwensspreekuur.

Wij willen graag weten hoe u dit ervaren heeft.

	Helemaal mee eens	Mee eens	Neutraal	Mee oneens	Helemaal mee oneens
Ik kreeg persoonlijke aandacht tijdens het gesprek	☐	☐	☐	☐	☐
Ik kreeg de mogelijkheid om zelf te beslissen of ik naar het kinderwensspreekuur toe ga	☐	☐	☐	☐	☐
Er werd rekening gehouden met mijn privacy	☐	☐	☐	☐	☐
Er werd uitgelegd waarom mijn kinderwens en het kinderwensspreekuur besproken werden	☐	☐	☐	☐	☐
Ik vind het goed dat er gevraagd werd of ik (nog) een kinderwens heb	☐	☐	☐	☐	☐

H2. Heeft u het fimpje over het kinderwensspreekuur bekeken?

U heeft, als het goed is, een link naar dit filmpje gekregen van het consultatiebureau.

- Ja ☐
- Nee ☐

H3. Wanneer vindt u dat u op een kinderwensspreekuur terecht moet kunnen?

- Zo snel mogelijk, binnen 2 weken ☐
- Binnen 2 maanden ☐
- Binnen 6 maanden ☐
- Binnen een jaar ☐
- Pas na een jaar ☐

H4. Vindt u dat uw partner betrokken moet worden bij het kinderwensspreekuur?

- Ja ☐
- Nee ☐
- Ik heb geen partner ☐

H5. Hoe vindt u dat u informatie moet krijgen over het kinderwensspreekuur?

	Ja	Misschien	Nee
Internet	☐	☐	☐
Bibliotheek	☐	☐	☐
Buurthuis	☐	☐	☐
Posters	☐	☐	☐
Informatiefolder	☐	☐	☐
Via de verloskundige/gynaecoloog	☐	☐	☐
Via de huisarts	☐	☐	☐
Via het consultatiebureau	☐	☐	☐
Via school	☐	☐	☐
Via werk	☐	☐	☐
Via vrienden/familie	☐	☐	☐

H6. Is er nog een andere manier waarop u informatie wilt krijgen over het kinderwensspreekuur?

Nee ☐

Andere ☐

Andere

H7. Naar welke zorgverlener zou u willen gaan voor advies over een volgende zwangerschap (kinderwensspreekuur)?

	Ja	Misschien	Nee
Huisarts	☐	☐	☐
Verloskundige	☐	☐	☐
Gynaecoloog	☐	☐	☐
Kinderarts	☐	☐	☐
Consultatiebureau arts	☐	☐	☐
Consultatiebureau verpleegkundige	☐	☐	☐

H8. **Is er nog een iemand anders waar u naar toe zou willen gaan voor advies over een volgende zwangerschap?**

Nee ☐

Andere ☐

Andere

H9. **Bent u eerder naar een kinderwensspreekuur geweest?**

Ja ☐

Nee ☐

H10. ***Dit is het einde van de vragenlijst.***

Bedankt voor het invullen van deze vragenlijst.

Over ongeveer een half jaar ontvangt u vragenlijst 2, een korte vragenlijst

Ruimte voor algemene opmerkingen:
