# Supplementary material for: Integrating interconception care in preventive child health care services: The Healthy Pregnancy 4 All program
Source: PLoS One. 2019 Nov 6;14(11):e0224427. doi: 10.1371/journal.pone.0224427 (PMC6834275; doi:10.1371/journal.pone.0224427)
Supplement: S7 Questionnaire — (PDF) [file pone.0224427.s009.pdf]

Questionnaire 2

Study into the pre-pregnancy clinics and the preparation for a subsequent pregnancy.

This questionnaire forms part of the **Healthy Pregnancy 4 All-2 Project**.

We are pleased that you want to complete this short second questionnaire. The results will contribute to improving the care prior to a pregnancy.

It will take you about 5 minutes to fill in the questionnaire.

There is space for you to write comments at the end of the questionnaire.

Please answer all the questions.

If you are in doubt, choose the answer that is closest to your situation.

Your answers will be treated confidentially and processed anonymously using a code.

The healthcare provider will not see the answers you write on this questionnaire.

There are 20 questions in this questionnaire

<u>General - registration</u>

What is your date of birth?

General - lifestyle and medical

Do you smoke?

- Yes, every day
- Yes, but only now and again
- No, I have never smoked
- No, but I did in the past

How much do you smoke on a normal day?

For example, how many *cigarettes* do you smoke per day?

Does your partner smoke?

- Yes
- No
- I do not have a partner

Are you taking folic acid tablets at the moment?

- Yes, every day
- Yes, but only now and again
- No

Your desire to have a child

The following questions apply to your situation at the moment.

When do you hope or expect to become pregnant again?

- I am pregnant at the moment
- Between now and 3 months
- Within 3 to 6 months
- Within 6 months to a year
- Within one to 2 years

- In 2 years or more
- I do not know yet when I want to become pregnant again
- I have been advised not to become pregnant again (for the time being)
- I do not want to become pregnant again

Do you or your partner use contraceptives?

- Yes
- No

Preparation for a pregnancy

Own Effort

You may already receive advice from a midwife, doctor or nurse before the pregnancy. We would like to know how difficult you find it to follow this advice.

	Very difficult	Fairly difficult	Fairly easy	Very easy
If you (were to) smoke: what would it be like for you to stop smoking?	◯	◯	◯	◯
How easy/difficult is it for you to take a pill (folic acid) every day?	◯	◯	◯	◯
How easy/difficult is it for you to visit a pre-pregnancy clinic?	◯	◯	◯	◯
How easy/difficult is it for you to discuss your desire for a child with a healthcare provider?	◯	◯	◯	◯

Statements about the baby's health and illness

Below we want to find out what you think you can do yourself to have a healthy baby

	I agree entirely	I agree	Neutral	I disagree	I disagree entirely
There is nothing that I can do to ensure that my baby is born healthy	◯	◯	◯	◯	◯
It is my job as a mother to ensure that my baby is born healthy	◯	◯	◯	◯	◯

	I agree entirely	I agree	Neutral	I disagree	I disagree entirely
There are few choices that I can make that will affect the health of my baby at his/her birth	○	○	○	○	○
There is a lot that I can do to ensure that my baby is born healthy	○	○	○	○	○
There are things that I can do before I become pregnant to ensure that my baby is born healthy	○	○	○	○	○

The Pre-Pregnancy Clinic

You have reached the second last page.

How did you hear about the existence of the Pre-Pregnancy Clinic?

- I was told about it at the Child Health Clinic
- Through posters at the Child Health Clinic
- Through a leaflet at the Child Health Clinic
- Other:

Did you go to the Pre-Pregnancy Clinic? *

- Yes
- No

When did you go to the Pre-Pregnancy Clinic? *

- About 0 to 2 months ago
- About 2 to 4 months ago
- About 4 to 6 months ago

Who at the Pre-Pregnancy Clinic did you have an appointment with?

- Doctor at the Child Health Clinic
- Nurse at the Child Health Clinic
- GP
- Midwife
- Gynaecologist
- Other

Can you also tell us who or which practice you visited?

What was the most important reason for you to decide that you <u>would</u> visit the pre-pregnancy clinic?

- I want(ed) to receive information / I want(ed) to prepare for a subsequent pregnancy
- Following advice from the Child Health Clinic
- Following advice from the midwife, gynaecologist or GP
- My partner wanted me to go
- Following advice from my family/friends
- The outcome of a previous pregnancy was not what I wanted
- I have a child with a condition
- Other:

The Pre-Pregnancy Clinic

You have reached the last page.

<u>Your experiences with the pre-pregnancy clinic</u>

You have indicated that you visited a pre-pregnancy clinic.

We would like to know how you experienced this.

	I <u>agree</u> entirely	I <u>agree</u>	Neutral	I <u>disagree</u>	I <u>disagree</u> entirely
I had the feeling that I could discuss everything	○	○	○	○	○
I felt free in my decision to follow the advice that I was given	○	○	○	○	○
My privacy was respected	○	○	○	○	○
My questions were answered	○	○	○	○	○
I was able to make an appointment at a time that suited me	○	○	○	○	○

	I agree entirely	I agree	Neutral	I disagree	I disagree entirely
I was able to visit the pre-pregnancy clinic run by the healthcare provider that I wanted to see	◯	◯	◯	◯	◯
The healthcare provider was well-informed	◯	◯	◯	◯	◯
I thought that the pre-pregnancy clinic was worthwhile	◯	◯	◯	◯	◯

	I agree entirely	I agree	Neutral	I disagree	I disagree entirely	Not applicable
My partner was included in the pre-pregnancy clinic	◯	◯	◯	◯	◯	◯
The 'Zwangerwijzer' questionnaire on the internet was worthwhile	◯	◯	◯	◯	◯	◯

What was the most important reason for you to decide not to visit the Pre-Pregnancy Clinic?

- I was unable to go to an appointment
- I dreaded going to the Pre-Pregnancy Clinic
- I could not get an appointment with the healthcare provider that I wanted to see
- I was not convinced about the benefit
- My partner did not think it was necessary
- I did not know what it would entail
- Other

Do you intend to visit the pre-pregnancy clinic in the future?

- Yes
- Maybe
- No

When do you think you will or might visit a Pre-Pregnancy Clinic?

- in approximately: months

This is the end of the questionnaire.

Thank you for filling in this questionnaire!
