# Supplementary material for: Integrating interconception care in preventive child health care services: The Healthy Pregnancy 4 All program
Source: PLoS One. 2019 Nov 6;14(11):e0224427. doi: 10.1371/journal.pone.0224427 (PMC6834275; doi:10.1371/journal.pone.0224427)
Supplement: S8 Questionnaire — (PDF) [file pone.0224427.s010.pdf]

Vragenlijst 2

Onderzoek naar kinderwensspreekuren en de voorbereiding op een volgende zwangerschap.

Deze vragenlijst hoort bij het project **Healthy Pregnancy 4 All-2** (Een Gezonde Zwangerschap voor Iedereen).

Wij zijn u dankbaar dat u ook deze korte tweede vragenlijst wilt invullen. De resultaten zullen bijdragen aan het verbeteren van de zorg voorafgaand aan een zwangerschap.

Het invullen van de vragenlijst duurt ongeveer 5 minuten

Aan het einde van de vragenlijst is er ruimte om opmerkingen te plaatsen.

Wij verzoeken u om alle vragen te beantwoorden.

Als u twijfelt, kies dan het antwoord dat het beste bij u past.

De gegevens uit deze vragenlijsten worden vertrouwelijk behandeld en anoniem met een code verwerkt.

De zorgverlener ziet de antwoorden die u heeft ingevuld in deze vragenlijst niet.

Er zijn 20 vragen in deze enquête

<u>Algemeen - registratie</u>

Wat is uw geboortedatum?

Algemeen - leefstijl en medisch

Rookt u?

- Ja, dagelijks
- Ja, af en toe
- Nee, ik heb nooit gerookt
- Nee, maar wel in het verleden

Hoeveel rookt u op een normale dag?

Bijvoorbeeld hoeveel *sigaretten* op een dag

Rookt uw partner?

- Ja
- Nee
- Ik heb geen partner

Gebruikt u nu foliumzuur tabletten?

- Ja, dagelijks
- Ja, af en toe
- Nee

Uw kinderwens

De volgende vragen gaan over uw situatie op dit moment.

Wanneer hoopt of verwacht u opnieuw zwanger te worden?

- Ik ben nu zwanger
- Binnen nu en 3 maanden
- Binnen 3 tot 6 maanden
- Binnen 6 maanden tot een jaar
- Binnen een jaar tot 2 jaar
- Over 2 jaar of langer

- Ik weet nog niet of ik opnieuw zwanger wil worden
- Ik heb het advies gekregen (voorlopig) niet opnieuw zwanger te worden
- Ik wil niet weer zwanger worden

Gebruikt u zelf of uw partner nu voorbehoedsmiddelen?

- Ja
- Nee

Voorbereiding op een zwangerschap

Eigen Inzet

Het kan zijn dat u al voor de zwangerschap van een verloskundige, arts of verpleegkundige adviezen krijgt. Graag willen wij weten hoe moeilijk u het vindt om die op te volgen.

	Heel moeilijk	Beetje moeilijk	Beetje makkelijk	Heel makkelijk
Als u (zou) rookt (roken): hoe is het voor u om te stoppen met roken?	○	○	○	○
Hoe is het voor u om elke dag een pilletje (foliumzuur) te slikken?	○	○	○	○
Hoe is het voor u om naar een kinderwensspreekuur te gaan?	○	○	○	○
Hoe is het voor u om u kinderwens te bespreken met een zorgverlener?	○	○	○	○

Uitspraken over gezondheid en ziekte van de baby

Hieronder willen wij weten hoe u denkt over wat je zelf kan doen om een gezonde baby te krijgen

	Helemaal mee eens	Mee eens	Neutraal	Mee oneens	Helemaal mee oneens
Er is niets wat ik kan doen om ervoor te zorgen dat mijn baby gezond geboren wordt	○	○	○	○	○
Het is mijn taak als moeder om ervoor te zorgen dat mijn baby gezond geboren wordt	○	○	○	○	○
Er zijn weinig keuzes die ik kan maken die van invloed zijn op de gezondheid van mijn baby bij de geboorte	○	○	○	○	○
Ik kan veel doen om ervoor te zorgen dat mijn baby gezond geboren wordt	○	○	○	○	○

	Helemaal mee eens	Mee eens	Neutraal	Mee oneens	Helemaal mee oneens
Er zijn dingen die kan doen voordat ik zwanger wordt om ervoor te zorgen dat mijn baby gezond geboren wordt	◯	◯	◯	◯	◯

Het Kinderwensspreekuur

U bent op de een-na-laatste pagina.

Hoe heeft u over het bestaan van het Kinderwensspreekuur gehoord?

- Het is mij op het consultatiebureau verteld
- Via de posters op het consultatiebureau
- Via de folder op het consultatiebureau
- Andere:

Bent u naar het Kinderwensspreekuur gegaan? *

- Ja
- Nee

Wanneer bent u naar het Kinderwensspreekuur gegaan?

- Ongeveer 0 tot 2 maanden geleden
- Ongeveer 2 tot 4 maanden geleden
- Ongeveer 4 tot 6 maanden geleden

Bij wie hebt u een afspraak voor het Kinderwensspreekuur gehad?

- Consultatiebureau arts
- Consultatiebureau verpleegkundige
- Huisarts
- Verloskundige
- Gynaecoloog
- Andere

Kunt u ook aangeven bij wie of welke praktijk u bent geweest?

Wat was voor u de belangrijkste reden om wel naar het kinderwensspreekuur te gaan?

- Ik wil(de) graag informatie/ ik wil(de) me goed voorbereiden op een volgende zwangerschap
- Op advies van het consultatiebureau
- Op advies van de verloskundige, gynaecoloog of huisart
- Mijn partner wilde dit graag
- Op advies van mijn familie / vrienden
- Ik heb een zwangerschap meegemaakt die anders is verlopen dat ik wilde
- Ik heb een kind met een aandoening
- Andere:

Het Kinderwensspreekuur.

U bent op de laatste pagina.

Uw ervaring met het kinderwensspreekuur

U hebt aangegeven dat u naar een kinderwensspreekuur bent geweest.

Wij willen graag weten hoe u dit ervaren heeft.

	Helemaal mee eens	Mee eens	Neutraal	Mee oneens	Helemaal mee oneens
Ik had het gevoel dat ik alles kon bespreken	○	○	○	○	○
Ik voelde mij vrij in mijn beslissing om de adviezen die ik kreeg op te volgen	○	○	○	○	○
Er werd rekening gehouden met mijn privacy	○	○	○	○	○
Mijn vragen werden beantwoord	○	○	○	○	○
Ik kon terecht voor een afspraak op het moment dat ik dat graag wilde	○	○	○	○	○
Ik kon naar het kinderwensspreekuur bij de zorgverlener die ik graag wilde	○	○	○	○	○

	Helemaal mee eens	Mee eens	Neutraal	Mee oneens	Helemaal mee oneens
De zorgverlener was goed geïnformeerd	◯	◯	◯	◯	◯
Ik vond het kinderwensspreekuur waardevol	◯	◯	◯	◯	◯

	Helemaal mee eens	Mee eens	Neutraal	Mee oneens	Helemaal mee oneens	Niet van toepassing
Mijn partner werd betrokken bij het kinderwensspreekuur	◯	◯	◯	◯	◯	◯
De Zwangerwijzer vragenlijst op internet was waardevol	◯	◯	◯	◯	◯	◯

Wat was voor u de belangrijkste reden om niet naar het Kinderwensspreekuur te gaan?

- Het lukte mij niet om naar een afspraak te gaan
- Ik zag op tegen het Kinderwensspreekuur
- Ik kon niet terecht bij de zorgverlener bij wie ik een afspraak wilde hebben
- Ik was niet overtuigd van de meerwaarde
- Mijn partner vond het niet nodig
- Ik wist niet goed wat ik me erbij voor moest stellen
- Andere

Bent u nog van plan om naar het Kinderwensspreekuur te gaan?

- Ja
- Misschien
- Nee

Wanneer denkt u dat u (eventueel) naar een Kinderwensspreekuur gaat?

- over ongeveer :   maanden

Dit is het einde van de vragenlijst.

Bedankt voor het invullen van deze vragenlijst!
